# The histone methyltransferase SDG8 mediates the epigenetic modification of light and carbon responsive genes in plants

**DOI:** 10.1186/s13059-015-0640-2

**Published:** 2015-04-19

**Authors:** Ying Li, Indrani Mukherjee, Karen E Thum, Milos Tanurdzic, Manpreet S Katari, Mariana Obertello, Molly B Edwards, W Richard McCombie, Robert A Martienssen, Gloria M Coruzzi

**Affiliations:** Department of Biology, Center for Genomics and Systems Biology, New York University, New York, NY 10003 USA; Cold Spring Harbor Laboratory, Cold Spring Harbor, New York, NY 11724 USA; School of Biological Sciences, The University of Queensland, St Lucia, Brisbane, QLD 4072 Australia; Instituto de Ingeniería Genética y Biología Molecular (INGEBI-CONICET), Vuelta de Obligado 2490 Piso 2, Buenos Aires, C1428ADN Argentina

## Abstract

**Background:**

Histone methylation modifies the epigenetic state of target genes to regulate gene expression in the context of developmental and environmental changes. Previously, we used a positive genetic screen to identify an *Arabidopsis* mutant, *cli186*, which was impaired in carbon and light signaling. Here, we report a deletion of the *Arabidopsis* histone methyltransferase *SDG8* in this mutant (renamed *sdg8-5*), which provides a unique opportunity to study the global function of a specific histone methyltransferase within a multicellular organism.

**Results:**

To assess the specific role of SDG8, we examine how the global histone methylation patterns and transcriptome were altered in the *sdg8-5* deletion mutant compared to wild type, within the context of transient light and carbon treatments. Our results reveal that the *sdg8* deletion is associated with a significant reduction of H3K36me3, preferentially towards the 3′ end of the gene body, accompanied by a reduction in gene expression. We uncover 728 direct targets of SDG8 that have altered methylation in the *sdg8-5* mutant and are also bound by SDG8. As a group, this set of SDG8 targets is enriched in specific biological processes including defense, photosynthesis, nutrient metabolism and energy metabolism. Importantly, 64% of these SDG8 targets are responsive to light and/or carbon signals.

**Conclusions:**

The histone methyltransferase SDG8 functions to regulate the H3K36 methylation of histones associated with gene bodies in *Arabidopsis*. The H3K36me3 mark in turn is associated with high-level expression of a specific set of light and/or carbon responsive genes involved in photosynthesis, metabolism and energy production.

**Electronic supplementary material:**

The online version of this article (doi:10.1186/s13059-015-0640-2) contains supplementary material, which is available to authorized users.

## Background

Epigenomic control modulates gene expression in response to environmental stimuli and developmental cues [1-6]. An important mechanism of this epigenomic control is covalent modification of histone proteins, such as histone methylation [7,8]. Histone modifications can be associated with activation or repression of gene expression depending on the specific amino acid substrate. For example, di- and tri-methylation of lysine (K) residues in the histone H3 tail at position K4 (H3K4me2 and H3K4me3) and tri-methylation of K36 (H3K36me3) are associated with actively expressed genes, while methylation at residues H3K9 and H3K27 (in particular H3K9me2 and H3K27me3) are associated with silenced genomic regions [8-10]. Interestingly, permissive histone modification (for example, H3K36me3) and repressive histone modification (for example, H3K27me3) were shown to have antagonistic roles in regulating gene activity [11]. This combinatorial nature of gene regulation via various histone modifications is collectively known as the ‘histone code’ [12].

The SET domain-containing group (SDG) histone methyltransferases (HMTs) are responsible for histone methylation and are conserved in yeast, animals and plants [13,14]. In single cell organisms like yeast, the global function of a specific HMT can be characterized by profiling the genome-wide histone methylation pattern in HMT loss-of-function mutants [15]. Such mutant studies greatly increased our understanding of specific HMTs, particularly their target preference. In mammals, global histone methylation profiling of SDG knockout/knockdown lines has been largely limited to animal cell lines, due to embryonic lethality of such mutants in transgenic animals [16,17]. In plants, mutations in specific SDG proteins result in detectable but non-lethal phenotypes, providing a unique opportunity to study the function of specific SDG proteins in the context of a multicellular organism. To date, *Arabidopsis* mutants in SDG HMTs have been probed at the level of transcriptome [18] and DNA replication patterns [18]. However, to our knowledge, the primary function of an SDG HMT - histone methylation - has not been studied at a genomic level in an *Arabidopsis sdg* mutant. Such a study should greatly improve our understanding of whether and how individual members of the SDG HMT family mediate methylation of histones associated with specific subsets of genes in the genome.

Here, we present an in-depth epigenomic analysis of *sdg8-5* (also known as *cli186*), an *Arabidopsis* mutant harboring a complete deletion of the HMT *SET domain-containing group 8* (*SDG8*). The *Arabidopsis* SDG8 is most similar to the H3K36 methyltransferase SET2 in yeast [13]. Despite the existence of 32 SDG HMT genes annotated in the *Arabidopsis* genome [13], loss-of-function mutations in *SDG8* show pleiotropic phenotypes, including early flowering [19-22], impaired pigment synthesis [23-25], enhanced branching [23-25], defective pathogen defense [7,26,27], altered hormone response [28], and altered touch response [29], suggesting a non-redundant role for SDG8 in *Arabidopsis*. The complete deletion mutant- *sdg8-5* characterized in this study - thus provides a great opportunity to characterize the global impact of *SDG8* deletion on histone methylation and gene expression in a multicellular eukaryote.

Previous analyses of the histone methylation role of SDG8 focused on single gene or gene family targets [7,20,22,24,26,30]. However, global histone methylation profiling of any *sdg8* allele, or any *sdg* mutant in *Arabidopsis* is still lacking. Furthermore, most of the *sdg8* mutant phenotypes were reported to be associated with H3K36 di- or tri-methylation [7,20,22,24,26,30], but some studies reported reduced histone H3K4 tri-methylation in *sdg8* alleles [21,29]. In this current study, we profiled the global histone methylation pattern of H3K4 and H3K36 in a *sdg8-5* mutant (a.k.a. cli186 [31]) compared with wild type. We discovered that SDG8 targets a subset of genes in the genome, preferentially the 3′ of the gene body, for H3K36 methylation. Moreover, this H3K36 methylation is associated with high-level gene expression in wild type, which is abrogated in the *sdg8-5* mutant. As a group, the SDG8 targets are enriched in carbon and/or light responsive genes and involved in specific biological processes such as defense response, primary metabolism, photosynthesis and energy metabolism. We also proposed a possible molecular mechanism involved in SDG8 target specificity.

## Results

### *sdg8-5* harbors a complete deletion of SDG8, a non-redundant member of the histone methyltransferase gene family in *Arabidopsis*

To isolate molecular components involved in integrating carbon (C) and light (L) signaling in plants, we previously designed a positive genetic screen using the carbon and light responsive *ASN1* promoter to identify a carbon and light insensitive mutant, *cli186* [31]. The *cli186* mutation was shown to be in a master regulatory hub essential for carbon and light regulation of a connected network of genes in energy, metabolism and photosynthesis in studies of etiolated *Arabidopsis* seedlings [31]. In this current study, we mapped the *cli186* mutation (a fast-neutron induced deletion) using Affymetrix ATH1 chips hybridized with genomic DNA [32] isolated from the *cli186* mutant versus wild type. Wild type here refers to the unmutagenized line containing pASN1-HPT2 transgene for the positive genetic screen described in [31], hereafter referred to as WT. This comparison revealed a deletion on chromosome 1, with a drastically reduced signal at the AT1G77300 locus in *cli186* compared with WT (Figure S1A in Additional file 1). The exact location of the deletion was then refined by PCRs with primers spanning the region surrounding AT1G77300. The deletion in *sdg8-5* spanned a 13.8 kb genomic sequence (Chr1:29,040,007-29,053,807), which contains AT1G77300 including its promoter, and a portion of the neighboring gene AT1G77310 (Figure S1B in Additional file 1). This initial analysis thus suggested AT1G77300, previously known as *SDG8* - a SET domain containing histone lysine methyltransferase, as a causal gene for the mutant phenotype.

To confirm that the deletion of *SDG8* is the causative mutation, we complemented the *cli186* mutant by transgenic introduction of *SDG8* with its native promoter (approximately 2 kb) and introns (Supplemental results in Additional file 1). Specifically, the carbon and light transcriptional repression of target gene *ASN1* in etiolated seedlings, which is significantly impaired in the *cli186* mutation compared with WT [31], is restored to wild-type level in the transgenic *cli186* plants complemented with the *SDG8* gene (Supplemental results, Figure S2 and Table S1 in Additional file 1). It is noteworthy that *SDG8* was also previously identified as the causal gene for early flowering in short days (*efs*) phenotype [19-21]. Similar to the *efs* allele (*fn210*), the *cli186* deletion allele also showed early flowering (Supplemental results, Figure S3 and Table S2 in Additional file 1). Additionally, both *cli186* and *fn210* (*efs*) alleles were abrogated in carbon and light repression of *ASN1* gene expression, as shown previously in etiolated seedlings [31] (Supplemental results and Table S3 in Additional file 1).

In summary, AT1G77300, which encodes a SET domain containing histone lysine methyltransferase called *SDG8*, is the causal gene of the C- and L-insensitive mutant phenotype of the *cli186* deletion mutant of *Arabidopsis* [31]. For the interest of clarity, we have renamed the *cli186* deletion mutant of *SDG8* as *sdg8-5*.

### SDG8 is associated with H3K36me3 marks on genes involved in specific metabolic and cellular processes

#### Identification of H3K36me3 hypomethylated genes in sdg8-5

The pleiotropic mutant phenotype of *sdg8* indicates that the encoded HMT performs a non-redundant function, even though the *Arabidopsis* SDG family contains 32 members [13]. *sdg8-5* therefore afforded us the opportunity to investigate the genome-wide histone methylation function of SDG8 [31]. Previous work has implicated SDG8 to be associated with H3K4 and H3K36 methylation marks based on analysis of single genes [7,20-22,24,26,30]. To probe the impact of *sdg8* deletion on the histone methylation pattern genome-wide, we performed chromatin immunoprecipitation sequencing (ChIP-Seq) using antibody against H3K36me3 or H3K4me3. The experiments were performed on 3-week-old light-grown plants, comparing *sdg8-5* and WT with a transient 2 h exposure to carbon and light treatments (see Materials and methods for details; Figure S4 and Table S4 in Additional file 1). Two independent biological replicates were analyzed with SICER [33] to identify genomic regions with differential histone marks between *sdg8-5* and WT.

**Figure 1:**
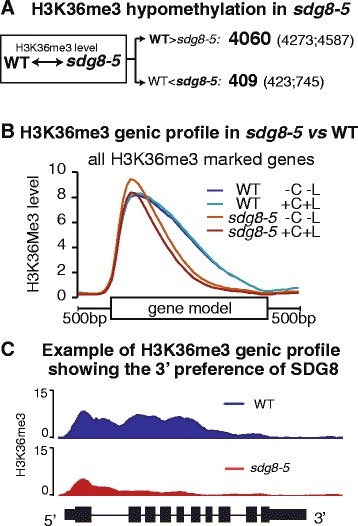
Altered global H3K36me3 profiles in *sdg8-5*, a complete deletion mutant of the histone methyltransferase SDG8. **(A)** The number of genes with differential H3K36me3 levels between the *sdg8-5* mutant and WT is listed. The major effect of the SDG8 deletion is the loss of H3K36me3 in 4,060 genes in the *sdg8-5* mutant. The numbers in parenthesis represent the number of differentially marked genes without or with a transient 2 h carbon and light treatment, while the number in bold is the common set between the two conditions. **(B)** The positional distribution of H3K36me3 on genic features was plotted and compared between *sdg8-5* and WT: for each gene with a significant H3K36me3 level, the gene model (based on phytozome annotation V7 of *Arabidopsis* genome TAIR10 (October 2011)) was divided into 40 bins, and 500 bp upstream and 500 bp downstream sequences were split into 10 bins each. The H3K36me3 level of each bin was calculated as the mean single nucleotide coverage from the ChIP library (calculated using BEDTools and RPM (Reads Per Million) normalized). The median H3K36me3 across all significantly marked genes is plotted (Enrichment level ChIP/Input >2, FDR <0.01, approximately 12,000 genes in WT and approximately 9,000 genes in *sdg8-5*). Since the deletion of SDG8 in *sdg8-5* causes a dramatic drop in the number of genes with H3K36me3 marks, the H3K36me3 level was further normalized to correct for the difference in genome coverage between WT and the *sdg8-5* mutant for the plot. Upon the deletion of SDG8, we observed a loss of H3K36me3 marks preferentially towards the 3′ of the gene model (B). **(C)** A gene example AT4G11960 where the H3K36me3 mark located towards the 3′ of the gene-coding region is lost in the *sdg8-5* mutant. Y-axis is the RPM normalized ChIP read counts of H3K36me3.

**Figure 2:**
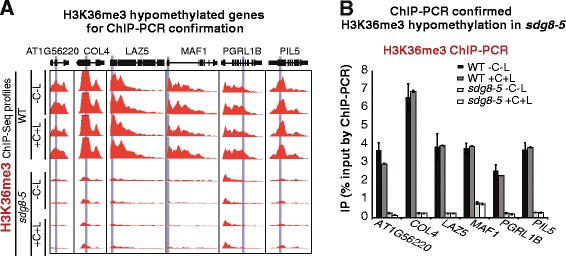
ChIP-PCR validation of H3K36me3 hypomethylation in *sdg8-5* compared with WT. ChIP-PCR was performed to validate the ChIP-Seq results of genes hypomethylated with H3K36me3 in *sdg8-5* compared with WT. **(A)** ChIP-PCR primers (blue columns) and H3K36me3 ChIP-Seq profiles (red plot) of these genes. **(B)** ChIP-PCR results confirmed the ChIP-Seq results. C, carbon; IP, immunoprecipitation; L, light. The error bars represent the standard error of the mean.

First, we observed a specific decrease of H3K36me3 marks in 4,060 genes in the *sdg8-5* mutant compared with WT, hereafter referred to as ‘hypomethylated genes’ ( false discovery rate (FDR) <0.05, fold change >2-fold; Figure 1A; for the gene list see Additional file 2). By contrast, few genes show different H3K4me3 marks between the *sdg8-5* mutant and WT (Figure S5A in Additional file 1). The hypomethylation of H3K36 residues was confirmed by independent ChIP-PCR assays of six exemplary genes, including the previously reported SDG8 target genes *MAF1* [22,34] for flowering control and *LAZ5* [26] for defense response (Figure 2A,B). Our genome-wide studies thus indicate SDG8 is a histone H3K36 methyltransferase of major influence. The H3K36me3 specificity of SDG8 is consistent with previous reports on *Arabidopsis sdg8* mutants based on single gene analysis [11,20,22,24,26,30,35], and with the role of its yeast ortholog [36].

#### Positional preference of H3K36 methylation by SDG8 within a gene

Next, we tested whether the deletion of SDG8 affects the positional profile of H3K36me3 on genic regions genome-wide. To determine this, we plotted the histone methylation level along gene models in *sdg8-5* and WT for all genes with a significant H3K36me3 level (Enrichment level ChIP/Input >2 and FDR <0.01; approximately 12,000 genes in WT and approximately 9,000 genes in *sdg8-5*; Figure 1B). We note that this cutoff eliminates genes that lose all detectable H3K36me3 in the *sdg8-5* mutant (since genes that completely lose H3K36me3 are not informative for determining positional preference). In WT, the H3K36me3 mark is most abundant at the 5′ of gene models and extends to the 3′ region (Figure 1B). A similar pattern of H3K36me3 distribution has been reported for plants including *Arabidopsis* [11,37] and maize [38,39], and this distribution is different from the H3K36me3 pattern in mammalian cells [9]. Interestingly, when *SDG8* is deleted, the genome-wide H3K36me3 pattern in the *sdg8-5* mutant is noticeably shifted towards the 5′ end, indicating a reduction in this mark in the 3′ portion of gene models (Figure 1B,C). By contrast, the positional distribution of H3K4me3 is unchanged between *sdg8-5* and WT (Figure S5B in Additional file 1). We also note that for some genes the H3K36me3 mark from the 5′ to 3′ is completely lost in the *sdg8-5* mutant (Figure 2A). Our data thus indicate that SDG8 is required to place the H3K36me3 mark along the gene body, with a bias towards the 3′ portion of the gene. This suggests a role for SDG8 in transcription elongation, rather than transcription initiation, similar to its yeast homolog [36]. The H3K36me3 mark towards the 3′ of genes could also be associated with mRNA processing (for example, exon/intron splicing), as indicated by its mammalian and yeast homologs [15,36,40]. The residual H3K36me3 in the 5′ end of the gene-coding regions in the *sdg8-5* mutant (Figure 1B,C) suggests that at least one or more other HMTs are responsible for depositing H3K36me3 to histones at the 5′ end of the gene-coding region, possibly involved in the initiation of the transcription process.

#### Functional analysis of the hypomethylated genes in the sdg8-5 mutant

We next examined whether SDG8 affects H3K36me3 associated with genes in specific biological pathways. Gene Ontology (GO) enrichment analysis [41] revealed that specific biological processes are significantly over-represented (FDR adjusted *P*-value <1E-6) in the 4,060 hypomethylated genes, including defense response, apoptosis, hormone signaling pathway, and pigment metabolic process, confirming previous single gene studies [25-28,35] (Table S5 in Additional file 1). We also identified several new biological processes as under the control of SDG8, including signaling cascade, phosphate/nitrogen/sulfate metabolism, primary metabolism and secondary metabolism, and responses to stimulus (including response to carbohydrate and response to light; Table S5 in Additional file 1). The latter result further validates our study as we identified the original *sdg8-5* mutant (formerly known as *cli186*) by screening for mis-regulation of carbon and light responses in *Arabidopsis* [31].

SDG8 was first identified as a regulator of flowering time [19]. In agreement with this, known flowering regulators *AGL22* and *MAF1* [42] are hypomethylated in H3K36me3 in the *sdg8-5* mutant compared with WT (Figure 2; Additional file 2). However, GO terms related to flowering control were not detected as significantly over-represented among the 4,060 hypomethylated genes in our study of the *sdg8-5* mutant, nor in previous transcriptome studies of other *sdg8* mutant alleles (*sdg8-1*, *sdg8-2* [22], *ccr1* [24] and *ashh2* [34]). This may possibly be due to the tissues and developmental stage assayed. Indeed, the flowering regulator *FLC*, previously reported to be down-regulated in some *sdg8* alleles (*sdg8-1* [20] and *sdg8-2* [22]), is not detected as differentially marked by H3K36me3 when *sdg8-5* is compared with WT in our ChIP-Seq, or when assayed by ChIP-PCR in plants at an earlier developmental stage (2-weeks) (Figure S6 in Additional file 1). This may be due to the dynamic nature of epigenetic control of *FLC* [11], as the H3K36me3 level of two other flowering time genes, *AGL22* and *MAF1* [42], are reduced in the *sdg8-5* mutant.

#### Genome clustering of the hypomethylated genes

In previous studies, genes under common epigenomic control are clustered in the genome [43,44]. Here, we tested whether the 4,060 hypomethylated genes form any chromosomal clusters using CROC (window size = 20 genes, *P*-value <0.05) [43]. Indeed, we found that 1,179 out of the 4,060 hypomethylated genes form 125 gene clusters in the genome. One such gene cluster on chromosome 4 containing 16 genes enriched with protein phosphorylation/phosphorus metabolic pathway (FDR <0.1) is shown for example (Figure S7 in Additional file 1).

#### SDG8 binding to the hypomethylated genes

Finally, if these 4,060 hypomethylated genes in the *sdg8-5* mutant are true direct targets of SDG8, we expect to detect the binding of SDG8 to at least some of these targets. We thus monitored SDG8-bound targets using an epitope tagged version of SDG8. To do this, SDG8 was fused to a hemagglutinin (HA) epitope and placed under the control of the native promoter of SDG8. This pSDG8::SDG8-HA construct was then transformed into the *sdg8-5* deletion mutant (see Materials and methods for details) to create an HA-tagged SDG8 transgenic line (*h*SDG8). This transgene was able to complement the *sdg8-5* mutant phenotype in early flowering (Table S6 in Additional file 1). We next tested whether the transgene could rescue the H3K36me3 hypomethylation in the *sdg8-5* mutant. To do this, we performed an H3K36me3 ChIP-Seq experiment to compare the H3K36me3 profile of *h*SDG8, WT and *sdg8-5*. In this assay, of the 4,567 genes that show H3K36me3 hypo*-*methylation in *sdg8-5* compared with WT, 93% (3,818 genes) show hyper*-*methylation of H3K36me3 in the *h*SDG8 plants compared with the *sdg8-5* mutant (with a cutoff FDR <0.05, fold change >2-fold for hypo/hyper-methylation). This suggests that HA-tagged SDG8 restores the wild-type level of H3K36me3 in *sdg8-5*. Indeed, only a few genes show significant differences in H3K36me3 levels between WT and *h*SDG8 (FDR <0.05, fold change >2-fold). Thus, the HA-tagged SDG8 transgene complements the H3K36me3 hypomethylation mutant phenotype in *sdg8-5*.

**Figure 3:**
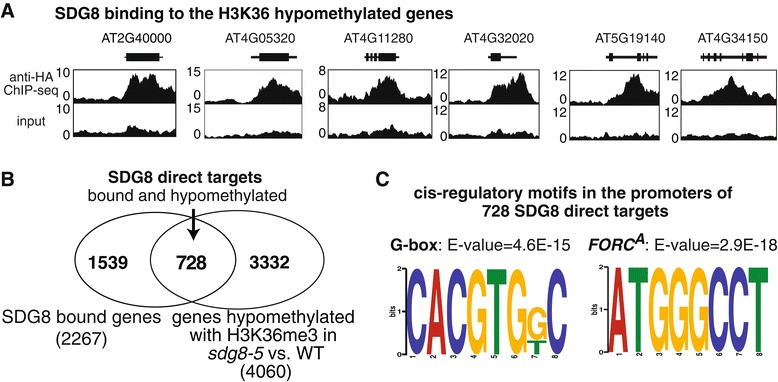
SDG8-binding of hypomethylated genes. **(A)** HA-tagged wild-type SDG8 was transformed into *sdg8-5* mutant background to allow detection of direct binding of SDG8 to the H3K36 hypomethylated genes by anti-HA ChIP-Seq. Examples of anti-HA ChIP-Seq profiles on H3K36 hypomethylated genes are shown, supporting that SDG8 directly binds to its functional targets. All genes are visualized with 5′ on the left, and 2 kb flanking intergenic regions or until the neighboring gene are also shown. **(B)** Intersection of 2,267 SDG8-bound genes and the 4,060 hypo-methylated genes in *sdg8-5* versus WT identified 728 SDG8-bound and hypomethylated targets (a.k.a. ‘SDG8 direct targets’). **(C)** Significantly over-represented *cis*-regulatory motifs G-box (bZIP binding motif) and *FORC* ^*A*^ are identified in the promoters of the 728 direct targets of SDG8 (bound by SDG8 and hypomethylated in *sdg8-5*).

Next, we used the *h*SDG8 transgenic plants to identify genes directly bound by SDG8 using anti-HA ChIP-Seq in light-grown 2.5-week-old plants. This uncovered 2,557 genomic regions bound by SDG8 (FDR <0.01 by SICER [33]), out of which 93% (2,381/2,557) co-localize with genic regions. This led to the identification of 2,267 genes that are bound to SDG8. A set of six representative SDG8-bound genes are shown in Figure 3A, which reveals preferential binding to the genic region. Of these SDG8-bound genes, 728 also show H3K36 hypomethylation in the *sdg8-5* mutant (Figure 3B; Additional file 2). These 728 SDG8 target genes represent a substantial (32%) and significant overlap (*P* < 4E-106 by hypergeometric distribution) between the genes bound by SDG8 (2,267) and genes whose associated H3K36 methylation is abrogated in the *sdg8-5* mutant (4,060) (Figure 3B). This confirms that the 4,060 hypomethylated genes are indeed enriched with direct targets of SDG8. We will focus our downstream analysis on these 728 SDG8-bound and hypomethylated genes, referred to as ‘SDG8 direct targets’ hereafter (Figure 3B).

### Transcriptome profiling of *sdg8-5* uncovers altered gene expression accompanied by altered epigenetic states

In a previous study, we showed that *sdg8-5* (formerly known as *cli186*) is impaired in carbon and light regulation of global gene expression, when assayed in etiolated seedlings [31]. Since SDG8 is an HMT involved in regulating multiple developmental processes in adult plants [22,23], we conducted a new transcriptome study to compare global gene expression in 3-week-old, light-grown *sdg8-5* plants and WT, with a 2 h transient treatment of light and/or carbon (see Materials and methods for details; Figure S4 in Additional file 1). A three-way ANOVA identified effects of carbon (C), light (L), genotype (G), and their interactions on global gene expression. This analysis shows that 2,158 genes and 1,923 genes, respectively, are expressed at significantly lower or higher levels in *sdg8-5* compared with WT (FDR <0.05 for factor G in three-way ANOVA; Figure S8 in Additional file 1; Additional file 3). Over-represented GO terms (FDR <0.01) were identified among these down- or up-regulated genes (mis-expressed genes) in *sdg8-5* (Figure S8 and Table S8 in Additional file 1). This GO term analysis is consistent with known functions of SDG8 in the defense response [26,27,35] and in pigment synthesis [24,25]. Interestingly, genes related to nitrogen metabolism are significantly enriched in these mis-expressed genes in *sdg8-5*. Specifically, the asparagine synthetase gene *ASN3* is more highly expressed in the *sdg8-5* mutant, while the glutamine synthesis genes *GLN1;3*, *GLN2*, and *GLN1;1* are down-regulated in *sdg8-5* mature plants*.* This suggests that alterations in H3K36me3 patterns shift nitrogen metabolism towards a ‘dark-adapted’ metabolic phenotype. Specifically, *sdg8-5* mutant plants convert glutamine into the more C-efficient nitrogen transport amino acid asparagine, used to transport N when C-skeletons are limiting [45].

In addition to the changes in gene expression caused by *SDG8* deletion, we also detected specific changes in the light regulation of gene expression in the *sdg8-5* mutant. Specifically, 127 genes are regulated by a G × L interaction (FDR <0.15 of G × L interaction in three-way ANOVA; Table S9 in Additional file 1), suggesting that their light responses are altered by the deletion of SDG8.

### Integration of epigenome and transcriptome data reveals that SDG8-dependent H3K36me3 correlates with level of gene expression

To test whether H3K36me3 hypomethylation in the *sdg8-5* mutant affects gene expression levels, we integrated the epigenome and transcriptome data assayed in the same experimental conditions (Figure S4 in Additional file 1).

In WT plants, we observed a positive correlation between the levels of H3K36me3 methylation and gene expression (Figure S9A in Additional file 1), which agrees with previous studies [4,38]. H3K36me3 has been reported to elevate gene expression levels by affecting other histone modification - for example, H3K4 acetylation through MRG domain protein [46]. Surprisingly, in the *sdg8-5* mutant, the correlation between level of gene expression and H3K36me3 methylation is disrupted (Figure S9A in Additional file 1). This reflects a reduction in the H3K36me3 level of the two bins of highest expressed genes. This disruption is specific to H3K36me3, as the positive correlation between levels of H3K4me3 and gene expression observed in WT is unaffected in the *sdg8-5* mutant (Figure S9B in Additional file 1).

To further study the relationship between H3K36 hypomethylation and gene expression, we compared the magnitude of H3K36me3 hypomethylation with the change of gene expression in *sdg8-5* versus WT (Figure S10A in Additional file 1). Indeed, the loss of H3K36me3 in *sdg8-5* is accompanied by a reduction of gene expression. In a binning analysis, the genes that exhibit the highest level of hypomethylation show the highest reduction in gene expression (Figure S10A in Additional file 1). In support of this, the 4,060 H3K36me3 hypomethylated genes (Additional file 2) have a highly significant overlap of 1,084 genes (*P* <1e-239) with the 2,158 down-regulated genes in *sdg8-5* (Additional file 3). Moreover, the 2,158 down-regulated genes in *sdg8-5* show a dramatic and specific drop in H3K36me3 levels compared with WT (Figure S10B in Additional file 1). By contrast, levels of H3K4me3 are unchanged for either the up-regulated genes or down-regulated genes in the *sdg8-5* mutant (Figure S10C,D in Additional file 1). In summary, the deletion of *SDG8* results in a specific loss of H3K36me3 marks and reduced gene expression.

### The direct targets of SDG8 are enriched with energy metabolism and photosynthesis genes

**Figure 4:**
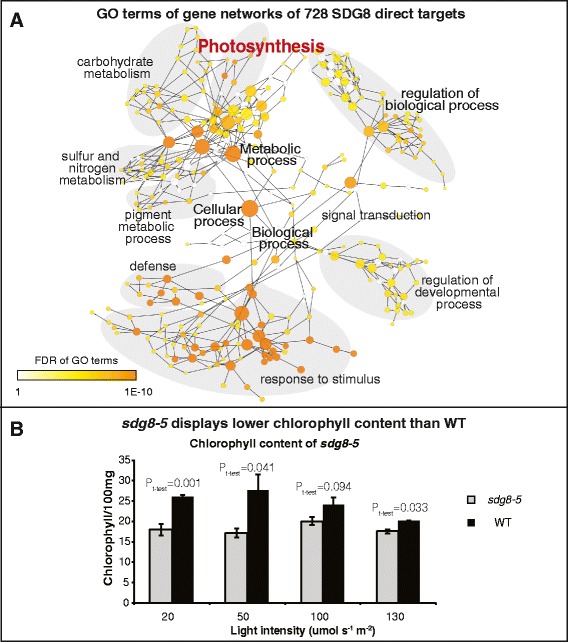
Biological processes enriched among the SDG8 direct targets and functional validation. **(A)** A network view of enriched biological processes among the 728 direct targets of SDG8 (bound by SDG8 and hypomethylated in *sdg8-5*). To generate this graph, gene regulatory network was first generated for the 728 SDG8 direct targets using Gene Network tool in VirtualPlant [50] with the *Arabidopsis* multinetwork interaction database. The regulatory edges were required to have one transcription factor binding site and gene expression correlation >0.7 calculated from the transcriptome in this study. The resulting gene network was then analyzed to generate the enriched biological process network shown in (A) (see Materials and methods). **(B)** Chlorophyll content in *sdg8-5* is significantly lower than that in WT, supporting the ‘photosynthesis’ pathway being regulated by SDG8. The error bars represent standard error of the mean.

To investigate the functional enrichment of the 728 direct targets of SDG8, a GO term analysis was performed, which revealed a significant enrichment (FDR adjusted *P*-value <1E-6) of biological process categories, including (1) response to abiotic/biotic stimulus; (2) defense response; (3) nutrient metabolism processes such as nitrogen and sulfur metabolism; (4) pigment metabolic processes and photosynthesis; (5) signal transduction such as protein phosphorylation; and (6) generation of precursor metabolites and energy and so on (for a complete list see Table S7 in Additional file 1). Interestingly, some of these biological processes are interlinked because defense response and nutrient metabolism are both sensitive to energy status [47,48], which is largely dependent on photosynthesis that requires pigment synthesis. Thus, SDG8 regulates genes involved in biologically related processes, possibly coordinating a system-wide reprogramming in cellular metabolic processes to balance energy demand and energy production. Indeed, analysis of the 728 SDG8 direct targets for significantly enriched KEGG (Kyoto Encyclopedia of Genes and Genomes) pathways [49] performed using the VirtualPlant platform [50] uncovered 38 ‘energy metabolism’ pathway genes as significantly enriched (FDR adjusted *P*-value <0.00646; Table S16 in Additional file 1). These 38 SDG8 target genes cover metabolic pathways in energy production (oxidative phosphorylation, photosynthesis, photosynthesis (antenna proteins) and carbon fixation in photosynthetic organisms) and energy use (nitrogen metabolism and sulfur metabolism) (Table S17 in Additional file 1). Overall, the GO term and KEGG pathway analysis of the SDG8 direct targets, is similar to that performed on the larger set of hypomethylated and down-regulated genes (Additional file 4; 1,084 genes; referred to as ‘functional targets’), which may also include indirect targets (Tables S10, S11 and S12 in Additional file 1). We validated these functional predictions of the SDG8 targets by showing that the deletion of *SDG8* in the *sdg8-5* mutant indeed causes a reduction in chlorophyll content in plants (Figure 4).

### The direct targets of SDG8 histone methyltransferase share a G-box binding site for bZIP transcription factors

In eukaryotes, gene regulation involves a complex interplay between transcription factors (TFs) and epigenetic regulators, which set the chromatin stage for TFs to activate or repress target genes. We thus investigated whether the direct targets of the HMT SDG8 share any common TF binding sites. To address this, we used MEME motif analysis [51] to first analyze the 500 bp upstream of the 728 SDG8 direct targets. Two *cis*-regulatory motifs were uncovered as significantly overrepresented in these SDG8 direct targets: the bZIP family binding motif G-box CACGTG (E-value = 4.6E-15) [52] and *FORC*^A^ motif TGGGC (E-value = 2.9E-18) [53] (Figure 3C). These two motifs are also significantly over-represented in the 500 bp upstream of the 1,084 functional targets of SDG8 (Figure S11 in Additional file 1).

The finding that the bZIP family binding motif G-box is enriched among the SDG8 targets suggests a functional connection between bZIP family TFs with the HMT SDG8. One speculative mechanism could be that a bZIP family TF binds to its targets through the G-box motifs, and then recruits SDG8 to these targets. Such a mechanism has been reported for bZIP11 and histone acetylation machinery [54]. Another possibility is that SDG8 modifies the chromatin states of targets of the bZIP family of proteins, which allows the binding of bZIP family protein to these targets to activate transcription. In either scenario, our result suggests an interplay between bZIP family TFs and SDG8 in gene regulation.

The transcription factor(s) associated with the other recovered *cis*-motif, the *FORC*^A^ motif (TGGGC; Figure 3C) is currently unknown [53]. The *FORC*^A^ motif (TGGGC) was reported as a *cis*-regulatory motif to integrate light and pathogen responses [53]. Both these GO terms were identified as over-represented functional groups among the SDG8 targets in our study, thus confirming these previous reports, and now connecting the *FORC*^A^ motif to histone modification by SDG8.

### The role of SDG8 in carbon and light response

In a previous study, the *sdg8-5* deletion mutant (previously named *cli186*) was impaired in the carbon and light transcriptional regulation of a connected network of genes in etiolated seedlings [31]. We thus investigated the relationship between SDG8 and carbon/light responses in this current study, where light-grown adult plants were treated with a transient 2 h carbon/light treatment, and profiled at both the transcriptome and H3K36me3 modification levels.

**Figure 5:**
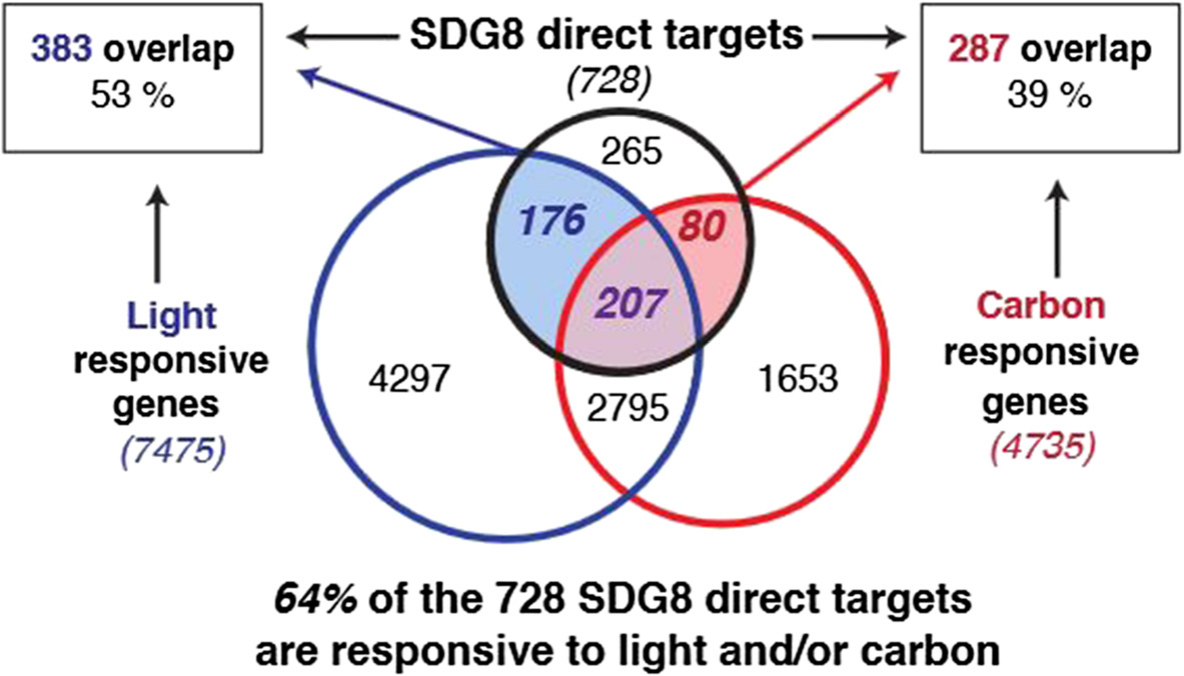
SDG8 targets are enriched in carbon and light responsive genes. The majority (64%) of the 728 direct targets of SDG8 (bound by SDG8 and hypomethylated in *sdg8-5*) are responsive to carbon, or light, or both.

Here, we found that SDG8 direct targets are enriched in genes responsive to light and carbon signals. In this study, we detected 4,735 genes that are transcriptionally regulated by the 2 h carbon (C) treatment (FDR <0.05 for C factor in three-way ANOVA), and 7,475 genes regulated by the 2 h light (L) treatment (FDR <0.05 for L factor in three-way ANOVA). Impressively, 64% of the 728 direct targets of SDG8 (463/728) are responsive to either carbon, light, or both signals (Figure 5). For light signaling, 53% of the 728 direct targets of SDG8 (*P*-value <4.2E-17 by hypergeometric distribution) are regulated by the 2 h light treatment (Figure 5). For carbon signaling, 39% of the 728 direct targets of SDG8 (*P*-value <1.2E-21 by hypergeometric distribution) are regulated by the 2 h carbon treatment (Figure 5). Similar results were found for the larger group of 1,084 functional targets of SDG8 (Figure S12A in Additional file 1). These results support that SDG8 plays a role in boosting H3K36me3 and expression levels of carbon/light responsive genes.

The *sdg8-5* mutant was previous reported to display impaired carbon and light gene regulation in etiolated seedlings [31]. In accordance with this, in our study of light-adapted plants with transient carbon and light treatment, 127 genes are significantly regulated by a Genotype (for example, *sdg8-5* versus WT) × Light interaction (FDR <15% of G × L interaction in ANOVA) (Table S9 in Additional file 1). Among these 127 G × L regulated genes, 57 genes (45%, *P*-value <4E-38 by hypergeometric test) belong to the 1,084 functional targets of SDG8 (Figure S12B in Additional file 1; Additional file 4). Additionally, 8 out of the 127 G × L regulated genes also belong to the 728 direct targets of SDG8 (Figure S13 in Additional file 1). This smaller but significant overlap (*P*-value <0.049 by hypergeometric test) indicates that the SDG8-dependent light response involves direct targets of SDG8. This is likely an underestimate due to false negative rates of SDG8 binding ChIP-Seq.

To follow up on this result, we tested whether SDG8 mediates any epigenetic response to light and carbon signals. We found that a group of 54 genes in WT gained higher H3K36me3 methylation levels in response to the 2 h carbon and light treatments (fold change >1.3, FDR <0.05; Table S13 in Additional file 1). These genes are significantly enriched (FDR <0.01) with biological processes ‘response to light stimulus’ and ‘carbon fixation’ (Table S14 in Additional file 1), suggesting that carbon and light indeed activate functionally relevant genes through an increase in permissive histone marks. Importantly, these 54 genes are significantly enriched (28/54, *P* < 2E-10 based on hypergeometric distribution with complete gene set as background) with genes dependent on SDG8 for H3K36me3 marks (that is, the 4,060 hypomethylated genes). Such a significant enrichment suggests that SDG8 plays a major role in mediating epigenomic responses to carbon and light signals. Indeed, in the *sdg8-5* mutant, a much smaller and different set of genes (only nine genes) show increased H3K36me3 level in response to the carbon and light treatment (fold change >1.3, FDR <0.05) (Table S13 in Additional file 1), with no over-represented biological processes. This result supports that the normal level of H3K36me3 accumulation in response to carbon and light treatment requires a functional SDG8 protein. Indeed, among the 54 genes that gain H3K36me3 in response to carbon/light in WT, but not in *sdg8-5*, 20.4% (11/54, *P*-value <1E-04 by hypergeometric test) belongs to the 1,084 functional targets of SDG8 (Figure S12B in Additional file 1). The overlap between the 54 genes and the 728 direct targets of SDG8 is much smaller (Figure S13 in Additional file 1), possibly caused by false negatives of SDG8 binding ChIP-Seq, or an indirect role of SDG8 in mediating the light response.

In summary, our analysis of the *sdg8-5* mutant reveals a specific role for SDG8 in maintaining the elevated H3K36me3 levels and gene expression levels of genes responsive to carbon and light signals. In addition, we showed that plants respond to the carbon and light signals at both the epigenetic and transcriptional levels, in part through SDG8.

## Discussion

Here, we showed that an *Arabidopsis* mutant impaired in carbon and light signal transduction from a prior genetic selection [31] was the result of a complete deletion of SDG8, an H3K36 methyltransferase*.* Thus, the *sdg8-5* mutant offered us the unique opportunity to study the genome-wide effect of one specific HMT in plants, and to address its global role in histone modification, gene expression and carbon and light signaling.

We discovered that 4,060 genes are specifically dependent on SDG8 to sustain normal levels of H3K36me3 marks on their associated histones. Therefore, *in vivo* global studies reported previously [22] and now enhanced by our ChIP-Seq data support SDG8 as a major H3K36 HMT in plants.

Our SDG8-binding data show that the H3K36me3 hypomethylated genes are significantly enriched in direct targets of SDG8. Specifically, we identified 728 direct SDG8 targets, which are bound by SDG8 and are also H3K36me3 hypomethylated in the *sdg8-5* deletion mutant. We focused on these 728 direct targets of SDG8 in our analysis. However, since dynamic interactions of SDG8 and its target genes could be missed [55], we also considered a larger set of ‘functional targets’ (1,084 genes that are hypomethylated and down-regulated). All functional analyses of these two sets of SDG8 targets were similar.

Our global analysis suggests that SDG8 affects the H3K36me3 histone mark associated with a specific set of genes involved in interrelated biological processes. These biological processes include primary metabolism (photosynthesis), nutrient metabolism (nitrogen and sulfur), and defense response. These specific biological processes are interlinked through energy metabolism. In fact, energy sources such as light and carbon are in high demand in these specific biological processes associated with SDG8 - for example, nitrogen metabolism (which is highly sensitive to the energy status in the cell [56,57]) and defense response [47]. Interestingly, we identified the two most significant *cis*-regulatory motifs in the promoters of the SDG8 targets as bZIP-binding motif and *FORC*^*A*^ motif. bZIPs are reported to integrate energy [57], light/carbon signaling and nitrogen metabolism [52,56,58,59], while the *FORC*^*A*^ motif is reported to integrate light and defense responses [53]. Thus, we posit that SDG8 is likely an important cog/integrator to deploy H3K36me3 to coordinate the transcription of genes involved in energy-sensitive processes genome-wide. As further proof, we show that the direct targets of SDG8 are largely carbon and light responsive, and that deletion of *SDG8* in the *sdg8-5* mutant impairs plant responses to carbon and light signals at both the epigenomic level and the gene expression level.

**Figure 6:**
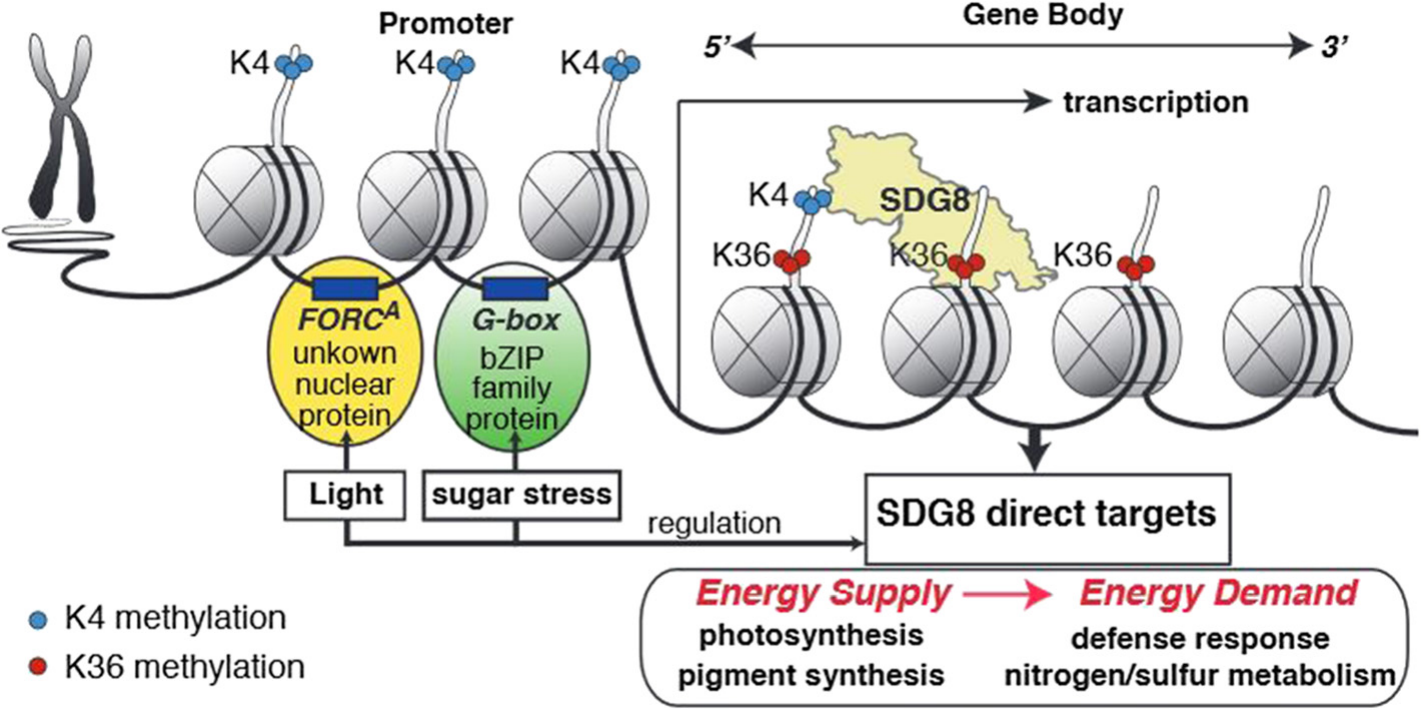
A model of H3K36me3 histone methylation and regulation by SDG8. SDG8 targets genes involved in cellular primary metabolism, nutrient metabolism and defense response by depositing permissive H3K36 methylation mark in the gene-coding region. Furthermore, SDG8 likely works in concert with transcription factors, such as the bZIP family, to poise and regulate genes responsive to light and energy levels genome-wide.

Finally, it is interesting to ask how SDG8 recognizes its specific target genes for epigenomic control. One possible mechanism is that SDG8 gets to its target genes through an interacting TF partner. It has been reported that SET domain proteins form protein complexes with TFs [60]. By analyzing the promoters of direct targets of SDG8, we identified bZIP family binding motif G-box and *FORC*^*A*^ motifs as over-represented among the SDG8 targets. This uncovers an interesting possibility that SDG8 may work with bZIP family TFs to regulate its targets. A similar mechanism was reported for bZIP11 recruitment of histone acetylation machinery to target genes [54]. Thus, SDG8 might regulate and work in concert with TFs, such as bZIP family TFs, to recognize its specific target genes (Figure 6).

## Conclusions

Our results support the notion that the H3K36 methyltransferase SDG8 is a central integrator of cellular energy metabolism in plants. They suggest that SDG8 boosts the permissive histone mark H3K36me3 and transcriptional levels of genes regulated by light and/or carbon. Collectively the SDG8 target genes are involved in cellular primary metabolism, photosynthesis, nutrient metabolism, and defense responses. Our model suggests the epigenetic marks by SDG8 possibly function to coordinate a broad genome-wide regulation of genes involved in energy supply and energy demand (Figure 6).

## Materials and methods

### Mapping of *cli186* (*sdg8-5*) deletion using ATH1 chips

To localize the site of the deletion in *cli186* (*sdg8-5*), Affymetrix ATH1 chips were hybridized with genomic DNA isolated from the *cli186* (*sdg8-5*) mutant and compared with WT (the unmutagenized line containing the ASN1-HPT2 transgene as described in [31]) following the protocol of [32] with two biological replicates.

### Construction of cli186-gSDG8 transgenic line

A T14N5 BAC clone containing the entire genomic sequence of At1g77300 (SDG8) was obtained from the Ohio State University Arabidopsis Biological Resource Center (ABRC). The full-length (12 kb) genomic region of At1g77300 was amplified with LA Taq using gD2_EFS (5′ TGGGCTCTTGTGAGGAGGCGGCCAAGTTACAAG 3′) and gU2_EFS primers (5′ GCGCGGGATATCCAGCAATGAGACGCTTCTTAAGC 3′). The full-length genomic-SDG8 fragment, which includes a 2 kb promoter (until the next gene), exons and introns, was cloned into pCR8/GW/TOPO vector. After verifying the insertion in the vectors, the insertions were cloned into a pMDC123 vector. The gSDG8-pMDC123 construct was used to transform *Agrobacterium tumefaciens* (strain GV3101). *A. tumefaciens*-mediated transformation of *cli186* (*sdg8-5*) was accomplished according to the floral dip protocol [61]. T1 seeds were surface sterilized and plated on MS medium supplemented with Kanamycin (50 μg/ml). The kanamycin-resistant plants were transferred to soil and allowed to set seed (T2). Transgenic lines that displayed a 3:1 ratio for kanamycin resistance in the T2 generation and that displayed 100% kanamycin resistance in the T3 generation were selected for further analysis. All experiments were performed using plants from the T4 to T6 generations.

### Plant growth for transcriptome assay, histone ChIP-Seq and ChIP-PCR validation

Plant tissues for transcriptome, histone ChIP-Seq and ChIP-PCR validation were grown independently following the same experimental process. WT and *sdg8-5* (previously named *cli186*) seeds were surface sterilized and imbibed in darkness for 2 days. Plants were then grown hydroponically inside a sterile Phytatray (Sigma-Aldrich, St Louis, MO, USA) on liquid Basal MS medium (GIBCO/Life Technologies, Grand Island, NY, USA; Formula 97-5068EC) supplemented with 1% sucrose and 2 mM KNO_3_ at a pH of 5.7. The phytatrays were kept under white light (50 μE m^−2^ s^−1^) in long-day cycle (16 h light/8 h dark) at 22°C for 3 weeks. After 3 weeks, the plants were transferred to liquid basal MS medium (GIBCO Formula 97-5068EC) supplemented with 0% sucrose and 2 mM KNO_3_ at a pH of 5.7 and the phytatrays were covered with aluminum foil (for light starvation) for 24 h. Plants were then treated with ±1% sucrose and ± light (70 μEin m^−2^ s^-1^) for 2 h. Shoots were flash-frozen in liquid nitrogen for RNA extraction. Shoots were also harvested and fixed with 1% formaldehyde for ChIP. Specifically for ChIP-PCR of flowering control genes, *sdg8-5* and WT were also sampled at 2-week-old stages, in addition to the 3-week-old stage, under the same growth conditions but without the carbon and light starvation and treatment.

### Histone ChIP-Seq of *sdg8-5* and WT

ChIP was performed according to [62] with two major modifications: chromatin was sonicated for 12 cycles (30 s high; 1 minute stop) using a Bioruptor sonicator (Diagenode, Seraing, Liege, Belgium) as described in [63] and then ChIP was performed using Dynabeads® Protein A (Life Technology, CA, USA) according to the manufacturer’s protocol. Anti-H3K4trimethylation antibody (Upstate,/Millipore, Billerica, MA, USA) and anti-H3K36me3 antibody (Abcam, Cambridge, MA, USA) were used. ChIP DNA (10 ng) and the input DNA (which was not immunoprecipitated and served as a background control) were used to construct Illumina paired-end sequencing library as described in [64] with adaptors P5 (5′ACACTCTTTCCCTACACGACGCTCTTCCGATCT) and P7-P (5′ phosphate-GATCGGAAGAGCGGTTCAGCAGGAATGCCGAG) and the following enrichment primers for 18 cycles of library enrichment: (1) forward primer, AATGATACGGCGACCACCGAGATCTACACTCTTTCCCTACACGACGCTCTTCCGATCT; and (2) reverse primer, CAAGCAGAAGACGGCATACGAGATCGGTCTCGGCATTCCTGCTGAACCGCTCTTCCGATCT. The libraries were sequenced on an Illumina Genome Analyzer IIX sequencer for 76 bp paired-end sequencing. Two independent biological replicates of ChIP-Seq were performed.

### Histone ChIP-seq data analysis of *sdg8-5* and WT

A minimum of 12 million 76 bp paired-end reads were generated for each library (Table S4 in Additional file 1). The raw sequencing reads were trimmed for quality and adaptor using an in-house Perl script and mapped to the *Arabidopsis* genome TAIR10 (Table S4 in Additional file 1) using Bowtie [65]. The lower percentage of chromosome mapped reads from the input DNA libraries, compared with the ChIP DNA libraries, was caused by a higher percentage of plastid genome mapping, while the ChIP DNA was depleted of plastid DNA due to a lack of histone in the plastid genomes. The chromosome mapped read pairs were then filtered to remove clonal fragments likely caused by PCR amplification in the library preparation. After this step, there are 10 million to 32 million fragments from each library remaining for the analysis of histone modification profiles using SICER according to the manual [33]. The genomic regions enriched with either H3K4me3 or H3K36me3 (referred to as ‘islands’) were determined by comparing the ChIP library with the input DNA library with SICER (SICER.sh) with the following parameters: fragment size was the median fragment size from Table S4 in Additional file 1; effective genome factor of 0.9; gap size of 200 bp; window size of 200 bp; redundancy threshold of 1. The ‘islands’ with an FDR <0.01 and enrichment level (ChIP/InputDNA) >2 are considered to be marked with H3K4me3 or H3K36me3, separately. The identified islands were then annotated with BEDTools [66] to highlight the genes associated with the islands. The differential analysis between mutant *sdg8-5* and WT, and between treated and untreated samples was also performed with SICER (SICER-df.sh). Window size and gap size were again set at 200 bp. For the pair-wise comparison between *sdg8-5* mutant and WT, differential islands were identified with a FDR cutoff <0.05 and a fold change of enrichment level (WT/*sdg8-5* or *sdg8-5*/WT) >2. For the pair-wise comparison between carbon and light treated samples and untreated samples, a FDR cutoff <0.05 and fold change of enrichment level (C and L treated/Untreated or Untreated/C and L treated) >1.3 was used, because a similar threshold was used for mild and transient treatment [4]. The resulting differential islands were then annotated with BEDTools [66] to identify the genes that are associated with significantly different H3K4me3 level or H3K36me3 level due to genotype/treatment difference.

To measure the correlation between two biological replicates, we applied three quality controls: (1) two biological replicates share at least 80% of the top 40% peaks ranked by FDR (Beta Cell Biology Consortium ChIP standards); (2) the Spearman correlation coefficiency of sequencing coverage is greater than 0.9 between the two biological replicates, calculated with three random selections of 300 kbp genomic regions [67]; (3) greater than 80% of the islands are shared between two biological replicates. Based on the three criteria, the two biological replicates were proven to be consistent. To enhance the confidence in true positives, we reported results only when they are true for both biological replicates - for example, a differentially methylated gene is reported only when it satisfies the statistical cutoff in both biological replicates.

### Affymetrix gene chip assays and data analysis

RNA (three sets of biological replicates) was isolated using RNeasy plant mini-kit (catalog number 74904) from Qiagen (Venlo, Limburg, Netherlands). The Affymetrix one-cycle cDNA synthesis kit was used to synthesize double stranded cDNA from 1 μg of total RNA. The cDNA was cleaned using the GeneChip Sample Cleanup Module (Affymetrix, 900371) and followed by biotin-labeling of the cRNA using the 3′ amplification reagent for IVT labeling (Affymetrix). The concentration and quality of cRNA was checked at A260/280 nm using the nanodrop. Finally, 8 μg of cRNA was used to hybridize the GeneChip *Arabidopsis* ATH1 genome array (from Affymetrix) at 42°C for 16 hours. Following the hybridization, the chips were washed and stained following the Affymetrix protocol. Finally the chips were scanned for further analysis.

The raw CEL files were normalized using the MAS5 package in the R environment [68]. A low expression level cutoff of 40 was applied to remove probes with extremely low expression level across all conditions, while most (21,552/22,810) probes were kept for the following statistical tests. A three-way ANOVA was performed to dissect the gene expression variation as caused by genotype, light, carbon, genotype × light, genotype × carbon, light × carbon, and three way interaction genotype × light × carbon in R. The raw *P*-value from the ANOVA was then adjusted for multi-testing error with FDR correction in R [69]. The FDR adjusted *P*-value was then used to select probes that are significantly affected by single factors, a binary interaction of two factors, or an interaction of all three factors. Only unambiguous probes mapped to a single gene were used. The clustering of gene expression patterns was performed with hierarchical clustering using MeV [70]. The over-represented GO term analyses presented in this study were performed with the BioMaps software in the VirtualPlant software platform [50] or AgriGO [71]. The significance test of overlaps between two gene sets was performed by hypergeometric distribution.

### *Cis*-regulatory motif analysis

The 500 bp upstream sequence from the ATG of the genes of interest was retrieved from the TAIR10 BLAST database (version 2010_10_28). MEME [51] was run on a local Unix machine to process the large input sequences with the following parameters: nmotifs = 15, minw = 5, maxw = 15, dna = TRUE, revcomp = TRUE.

### ChIP-PCR validation of H3K36me3 hypomethylation

The quantitative PCR primers were designed using IDT DNA tool kit for nine genes (Table S15 in Additional file 1): i) six genes (AT1G56220, COL4, LAZ5, MAF1, PGRL1B, and PIL5) from the 4,060 hypomethylated gene list (Additional file 2); ii) FLC; iii) two reference genes (RNA helicase [72], actin [35,73]). Four primers spanning the genic region of FLC were designed (Figure S6 in Additional file 1). For the other genes, one pair of primers was designed to the peak of the H3K36me3 in the gene body (Figure 2A; for the sequences of primers see Table S15 in Additional file 1). The amplification efficiency of all primers was determined using standard curve (Efficiency = 90% to 110%). The ChIP was performed as described in the ‘Histone ChIP-seq of *sdg8-5* and WT’ section. The ChIP-PCR was performed with LightCycler® FastStart DNA MasterPLUS SYBR Green I system (Roche, Basel, Switzerland) in Light Cycler 480 II (Roche). The percentage input was calculated by first normalizing ChIP to the input DNA as 2^(input Ct-ChIP Ct)^ × Input dilution factor as described in [73], and then normalized to reference genes. For quality control, fold enrichment was also calculated as 2^(no antibody Ct-ChIP Ct)^ to make sure that the fold enrichment over no antibody control is at least greater than 3 (while majority fold enrichments over no antibody control are greater than 10). Three biological replicates were assayed for all genes with the 3-week-old samples. Two biological replicates were assayed for FLC with the 2-week-old samples.

### Global binding profile of *h*SDG8

#### Transgenic plants

Genomic DNA of SDG8 was cloned from *Arabidopsis* BAC clone T14N5 using primers (ACTGTTGAGCTTCTTCTCTAAAGTTAGATT) and (CACCGCGCGGGATATCCAGCAATGAGACGCTTCTT), which amplifies the 2 kb upstream promoter, 5′ UTR, exons and introns until the stop codon, into pENTR/D-TOPO vector (Life Technologies). This insert was then introduced into pEARLEY301 binary vector [74] by Gateway cloning to produce a carboxy-terminal HA-tagged SDG8 (*h*SDG8), with its native promoter and exon-intron structure. The pEARLEY301 binary vector was then transformed into *sdg8-5* using the floral dip method [61]. The *h*SDG8 T1 transgenic plants were selected by BASTA resistance and confirmed by PCR genotyping.

#### ChIP-Seq

Positive transgenic *h*SDG8 plants were used for the HA-tagged SDG8 binding profiling by ChIP-Seq, where an anti-HA antibody (Abcam) was used to pull down the HA-tagged SDG8 in chromatin samples prepared from the T2 generation, which is segregating with a 3:1 ratio of transgenic versus non-transgenic plants. The plants were grown in 1% agar plates with 1× Basal MS medium (GIBCO Formula 97-5068EC) supplemented with 1% sucrose, 2 mM KNO_3_ and 0.5 g/L NaMES at a pH of 5.7 for 2.5 weeks under 16 h 130 uE m^−2^ s^−1^ light/8 h dark cycle at 22°C. ChIP-Seq was performed using shoots as described in the ‘Histone ChIP-seq of sdg8-5 and WT’ section, except for the following modifications: 1) anti-HA antibody (Abcam) was used; 2) barcoded adaptors and enrichment primers (BiOO Scientific, Austin, TX, USA) were used for preparing Illumina Hi-Seq compatible ChIP-Seq libraries. In addition, *sdg8-5* and WT were also grown together with *h*SDG8, and the global H3K36me3 profiling of *h*SDG8, *sdg8-5* and WT were performed by anti-H3K36me3 ChIP-Seq, using Illumina Hi-Seq compatible barcoded adaptors and enrichment primers (BiOO Scientific), to validate if *h*SDG8 could complement the *sdg8-5* mutant phenotype of H3K36me3 hypomethylation. Pooled barcoded libraries were sequenced on an Illumina HiSeq platform for 100 cycles in paired-end configuration (Cold Spring Harbor Lab, NY, USA). ChIP-Seq data analysis was performed as described in the ‘Histone ChIP-seq data analysis of sdg8-5 and WT’ section.

### Chlorophyll measurements

For chlorophyll measurements, *sdg8-5* and WT plants were surface sterilized and planted in 1% agar plates with 1× Basal MS medium (GIBCO Formula 97-5068EC) supplemented with 1% sucrose, 2 mM KNO_3_ and 0.5 g/L NaMES at a pH of 5.7. The plants were first vernalized at 4°C for four days, and then grown at 22°C under 16 h light/8 h dark cycle with light intensity of 20 μE m^−2^ s^−1^, 50 μE m^−2^ s^−1^, 100 μE m^−2^ s^−1^ and 130 μE m^−2^ s^−1^ separately for two weeks. Generally, four biological replicates were assayed, while nine plants were pooled for each replicate, except for light intensity of 130 μE m^−2^ s^−1^. For 130 μE m^−2^ s^−1^ , two biological replicates of *sdg8-5* and three biological replicates of WT were sampled, while each biological replicate is a pool of six seedlings. Only the shoots were collected for measuring chlorophyll fluorescence and biomass. The chlorophyll fluorescence was measured as described in [75]. Briefly, 500 μl of N,N-dimethyl-formamide was used to extract chlorophyll at 4°C in dark overnight, and then A_666_, A_647_, and A_603_ was measured using Nanodrop (Thermo Scientific) to calculate chlorophyll content, normalized to biomass as described in [75].

### Enriched GO term network

To generate Figure 4A, gene regulatory network was first generated for the 728 SDG8 direct targets using Gene Network tool in VirtualPlant [50] with the *Arabidopsis* multinetwork interaction database. The regulatory edges were required to have one transcription factor binding site and gene expression correlation >0.7 calculated from the transcriptome in this study. The resulting gene network was then analyzed using BiNGO [76] to generate the enriched biological process network.

### Data access

The ChIP-Seq data generated in this study have been deposited in the NCBI Sequence Read Archive (SRA) with accession number PRJNA265379. The transcriptome data generated in this study was deposited in the NCBI Gene Expression Omnibus (GEO) with accession GSE62435.

## Additional files

Additional file 1:
**A pdf file that contains the Supplemental methods, Supplemental results, Supplemental figures S1 to S13, and Supplemental tables S1 to S17.**
Additional file 2:
**A table listing the 4,060 hypomethylated genes in** ***sdg8-5*** **compared with WT and 728 direct targets.**
Additional file 3:
**A table listing the genes up-regulated or down-regulated in** ***sdg8-5*** **compared to WT.**
Additional file 4:
**A table listing the functional targets of SDG8.**

## Abbreviations

ANOVA: analysis of variance
C: carbon
ChIP-Seq: chromatin immune-precipitation sequencing
Cli: carbon and light insensitive
FDR: false discovery rate
GO: Gene Ontology
H3K36me3: histone H3 lysine 36 tri-methylation
H3K4me3: histone H3 lysine 4 tri-methylation
HA: hemagglutinin
HMT: histone methyltransferase
KEGG: Kyoto Encyclopedia of Genes and Genomes
L: light
SDG: SET domain-containing group
TF: transcription factor
UTR: untranslated region
WT: wild type
