# Supplementary material for: The histone methyltransferase SDG8 mediates the epigenetic modification of light and carbon responsive genes in plants
Source: Genome Biol. 2015 Apr 19;16(1):79. doi: 10.1186/s13059-015-0640-2 (PMC4464704; doi:10.1186/s13059-015-0640-2)
Supplement: Additional file 1: — A pdf file that contains the Supplemental methods, Supplemental results, Supplemental figures S1 to S13, and Supplemental tables S1 to S17. [file 13059_2015_640_MOESM1_ESM.docx]

**Additional File 1. Supplemental Results, supplemental methods, supplemental figures and supplemental tables.**

**Supplemental Results**

*Complementation of cli186 mutant (now renamed sdg8-5) phenotype with SDG8/ AT1G77300*

To confirm that the deletion of the *SDG8/AT1G77300* gene is indeed the cause of misregulation of *ASN1* in *cli186*, a full-length genomic SDG8 fragment (gSDG8) including the promoter and the 3’-UTR was introduced into the *cli186* mutant, and the molecular and growth phenotypes of the transgenic *cli186* plants containing the full-length SDG8 gene (cli186-gSDG8) were compared to WT (the unmutagenized line containing the pASN1::HPT2 transgene as described in [1], hereafter referred to as WT). In our previous studies [1], it was shown that the WT with pASN1::HPT2 reporter is hygromycin-sensitive in the presence of C and L, as the *ASN1* promoter is repressed by C and L (Fig. S2A). By contrast, the *cli186* mutant is hygromycin-resistant due to de-repression of the *ASN1* promoter (Fig. S2A) [1]. Several single-insertion homozygous lines of the cli186-gSDG8 transgenic plants were tested for the growth-phenotype on the Hygromycin plates. The complementation studies show that the gSDG8 transgene was able to restore the hygromycin-sensitive phenotype in the *cli186* mutant, similar to the hygromycin sensitivity observed in WT (Fig. S2A), further supporting our finding that SDG8 mediates the repression of *ASN1*. A similar hygromycin growth phenotype was observed when plants were grown in the darkness (Fig. S2B&C).

*Comparison of the mutant phenotype between cli186 (now renamed as sdg8-5) and another sdg8 allele fn210*

We next examined whether C and L repression of *ASN1* observed in *cli186* [1] is also abrogated in a previously identified fast neutron allele of SDG8 named *fn210* [2]. Indeed, the derepression of *ASN1* in *fn210* is comparable to that observed in *cli186* (Table S3). Thus, the C and L repression of *ASN1* mRNA is abrogated in both the *cli186* mutant and the *fn210* allele of *SDG8*. To further confirm that *cli186* is indeed a new allele of *fn210*, we tested whether *cli186* exhibits a similar early-flowering phenotype in short-days and long-days, as does the *fn210* mutant [2, 3]. Both *cli186* and *fn210* mutants flowered earlier than their respective WT when grown in short-days (Fig. S3). In short days, the number of leaves prior to flowering in both mutants (*cli186* and *fn210*) was about 7 leaves (~32 days), compared to 11 leaves in the respective wild-types (~43 days) (Table S2). In long-days, both *cli186* and *fn210* also exhibited a similar early flowering phenotype (Table S2).

**Supplemental Methods**

*Quantitative-PCR*

Total RNA was extracted from frozen samples using Trizol according to the manufacturer’s protocol (Invitrogen). Total RNA was treated with RNase-free DNase I to remove residual genomic DNA present in the samples. cDNA was prepared from total RNA according to the manufacturer’s instructions (Invitrogen). cDNA was diluted 1:5 for real time RT-PCR. Primer sets corresponding to the Arabidopsis ASN1 gene and to the Arabidopsis CLAT, the house-keeping gene were designed using Primer3 software. BLASTN searches were performed with the individual primer sets to confirm that had amplified only the specific gene of interest. Primers were designed so that they amplify ~150 bp region in length and were of same melting temperature.

*Hygromycin resistance test*

Seeds were sterilized and sown in plates with Basal MS media supplemented with 0.5% Sucrose, 2 mM KNO_3_ and 15 ug/ul Hygromycin; pH 5.7. Plates were positioned upright in growth chambers illuminated with 50 uE in m^-2^s^-1^ white light. Photos were taken after 3 weeks of growth in Long-day (16h Light /8h Dark) growth condition. For etiolated experiments, plates were kept in dark and photos were taken after 10 days of growth.

**Supplemental Reference**

1. Thum KE, Shin MJ, Gutiérrez RA, Mukherjee I, Katari MS, Nero D, Shasha D, Coruzzi GM: **An integrated genetic, genomic and systems approach defines gene networks regulated by the interaction of light and carbon signaling pathways in Arabidopsis**. *BMC Syst Biol* 2008, **2**:31.

2. Kim SY: **Establishment of the Vernalization-Responsive, Winter-Annual Habit in Arabidopsis Requires a Putative Histone H3 Methyl Transferase**. *PLANT CELL ONLINE* 2005, **17**:3301–3310.

3. Zhao Z, Yu Y, Meyer D, Wu C, Shen W-H: **Prevention of early flowering by expression of FLOWERING LOCUS C requires methylation of histone H3 K36**. *Nat Cell Biol* 2005, **7**:1156–1160.

4. Bailey TL, Boden M, Buske FA, Frith M, Grant CE, Clementi L, Ren J, Li WW, Noble WS: **MEME SUITE: tools for motif discovery and searching**. *Nucleic Acids Res* 2009, **37**(Web Server issue):W202–208.

5. Du Z, Zhou X, Ling Y, Zhang Z, Su Z: **agriGO: a GO analysis toolkit for the agricultural community**. *Nucleic Acids Res* 2010, **38**(Web Server issue):W64–70.

6. Katari MS, Nowicki SD, Aceituno FF, Nero D, Kelfer J, Thompson LP, Cabello JM, Davidson RS, Goldberg AP, Shasha DE, Coruzzi GM, Gutiérrez RA: **VirtualPlant: A Software Platform to Support Systems Biology Research**. *Plant Physiol* 2010, **152**:500–515.

7. Ogata H, Goto S, Sato K, Fujibuchi W, Bono H, Kanehisa M: **KEGG: Kyoto Encyclopedia of Genes and Genomes**. *Nucleic Acids Res* 1999, **27**:29–34.

**Supplemental Figures**


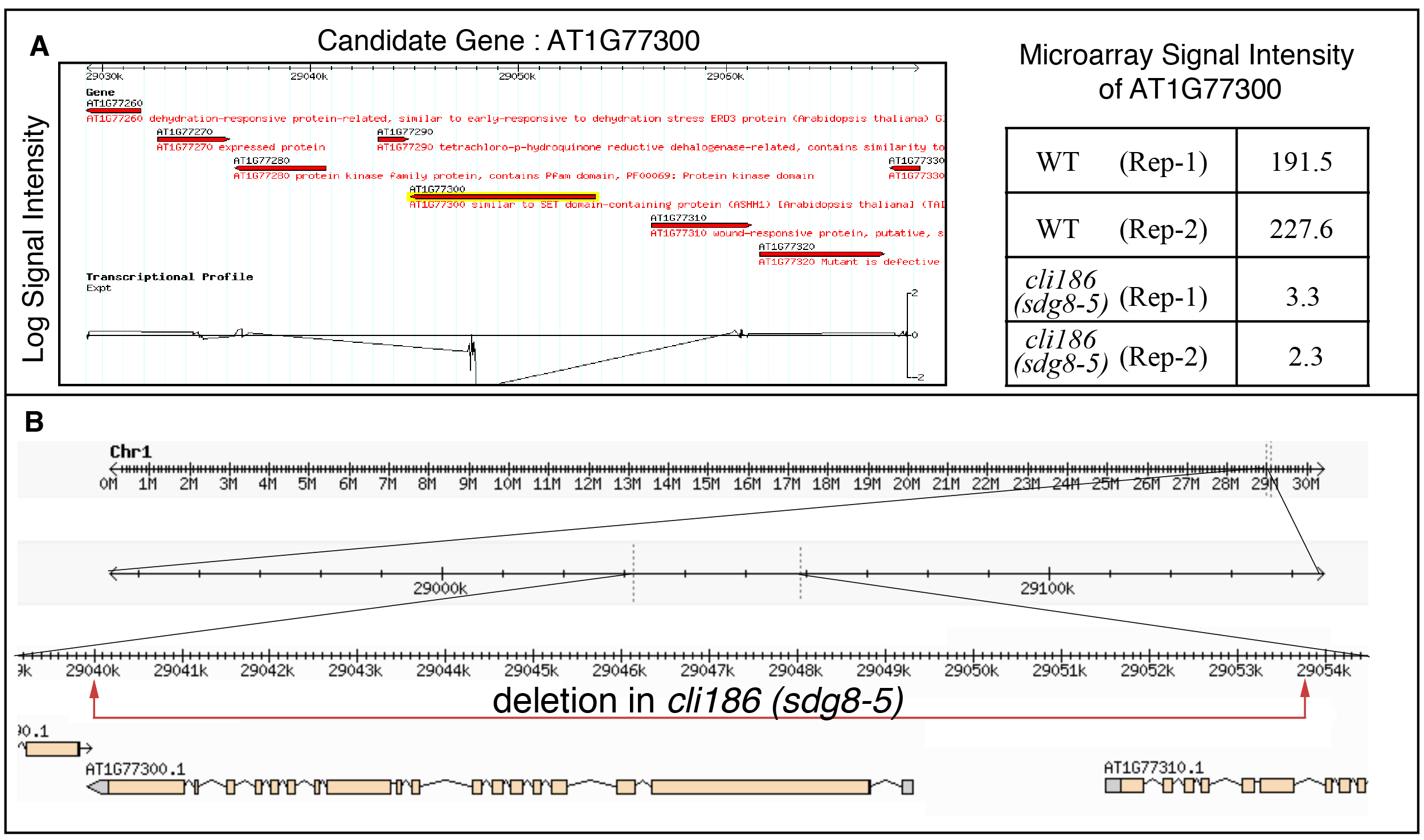


**Fig. S1 Mapping of deletion in *cli186 (*now renamed *sdg8-5).*** (A) The deletion in *cli186* was mapped to the genomic region around AT1G77300 using the ATH1 microarray. (B) Fine mapping with PCR primers spanning the AT1G77300 locus suggests that the deletion in *cli186* contains the whole genic region of AT1G77300 plus its promoter, as well as the first 6 exons of the neighboring gene AT1G77310.


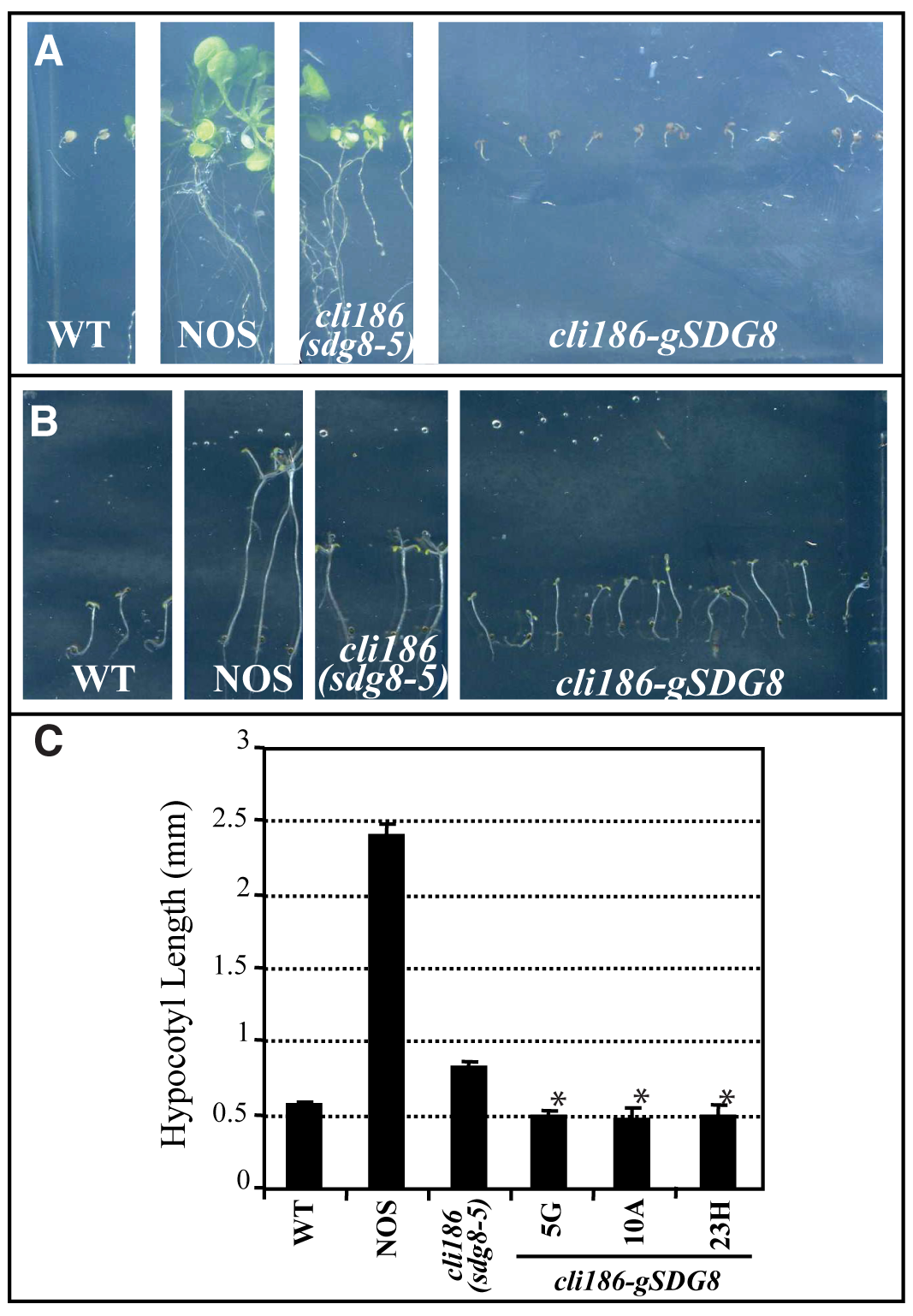


**Fig. S2. Complementation of *cli186* mutant (now renamed *sdg8-5*) phenotype by SDG8 transgene.** The misregulation of reporter pASN1::HPT in *cli186* mutant is rescued by SDG8 transgene in both light grown (A) and etiolated (B) growth condition, assayed as described in [1] and in supplemental method. The hypocotyl length of the seedlings in the etiolated conditions is shown (C). Controls consist of a 'wild-type' (WT) unmutagenized line containing the pASN1::HPT2 transgene and a transgenic line (NOS) containing the HPT2 transgene driven by a NOS promoter, allowing for constitutive expression of the HPT2 gene.


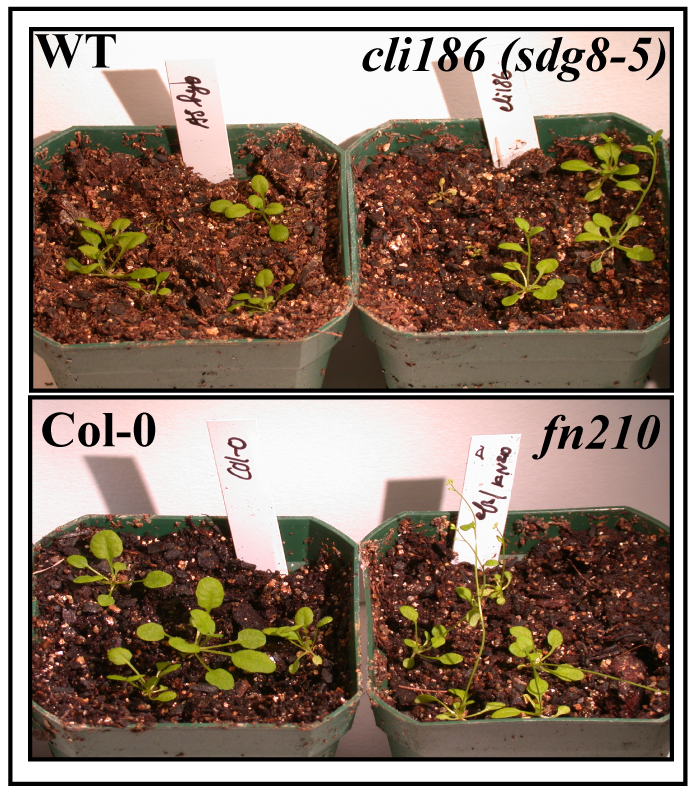


**Fig. S3** Early flowering phenotype of *cli186* mutant (now renamed *sdg8-5*) is comparable to the previously reported SDG8 mutant *fn210*.


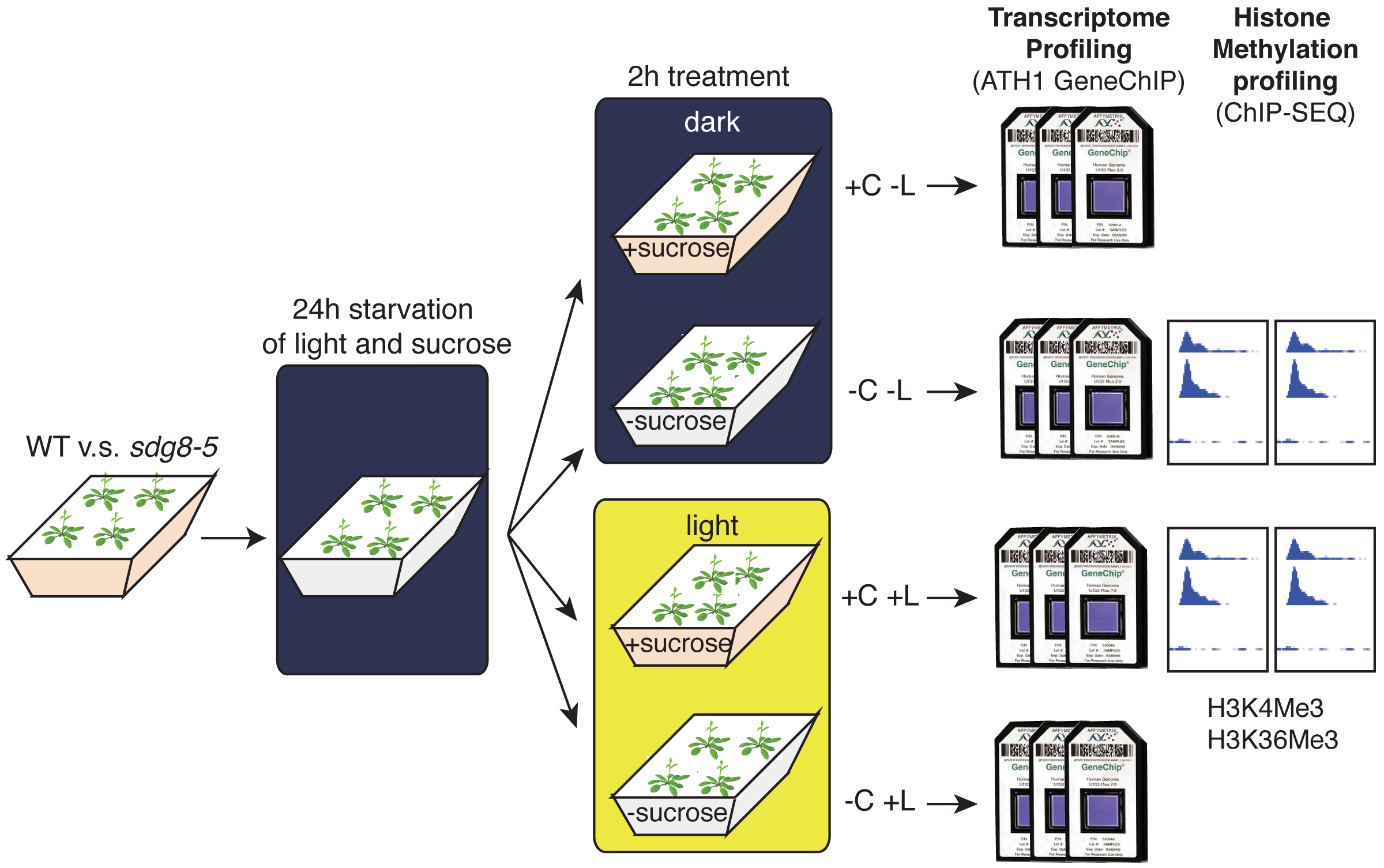


**Fig. S4** Experimental Scheme of the histone methylation ChIP-Seq and transcriptome profiling of *sdg8-5* and WT, in carbon (C) and/or light (L) treated samples and untreated controls.

**
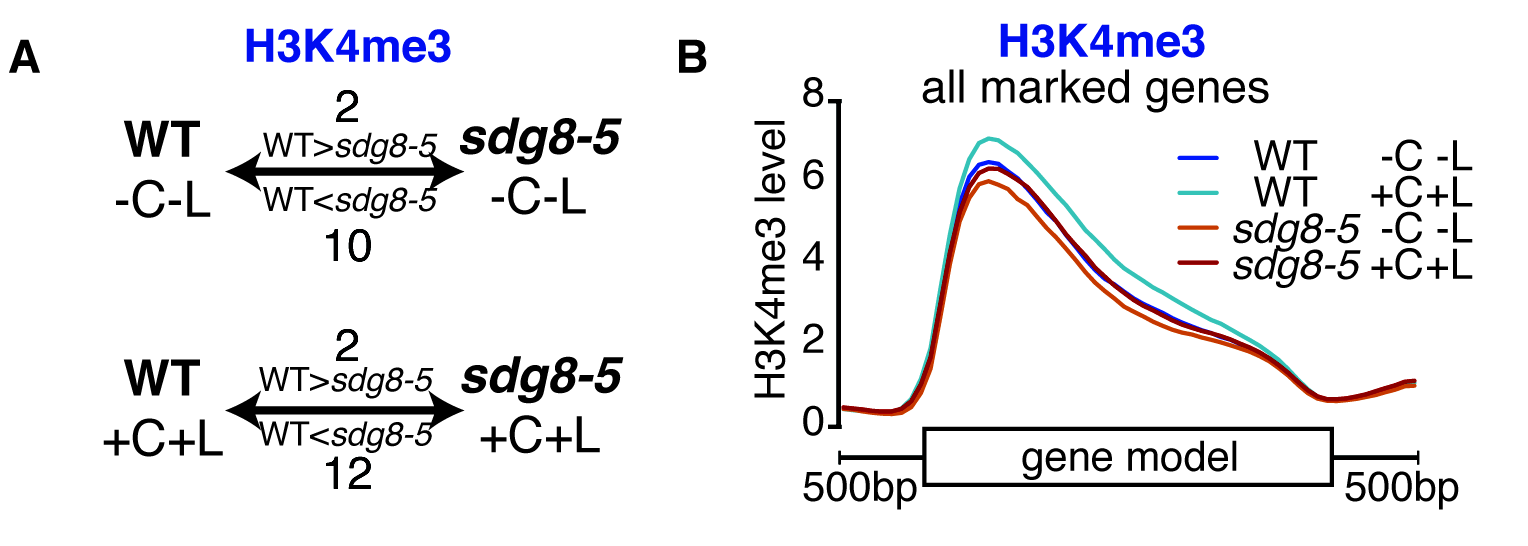
**

**Fig. S5 H3K4me3 profile is comparable between *sdg8-5* and WT.** (A) Number of genes with differential histone mark H3K4me3 between the *sdg8-5* mutant and WT is listed. (B) The positional distribution of H3K4me3 on genic features was plotted: First, for each gene, the gene model (based on phytozome (www.phytozome.net/) annotation V7 of Arabidopsis genome TAIR10, accessed Oct 2011) was divided into 40 bins, and 500bp upstream and 500bp downstream sequences were split into 10 bins each. H3K4me3 level of each bin was then calculated as the mean RPM-normalized single nucleotide coverage in the ChIP library within the bin. The median H3K4me3 level across all significantly marked genes (Enrichment level ChIP/Input >2, FDR<0.01, ~9,000 genes) is plotted.

**
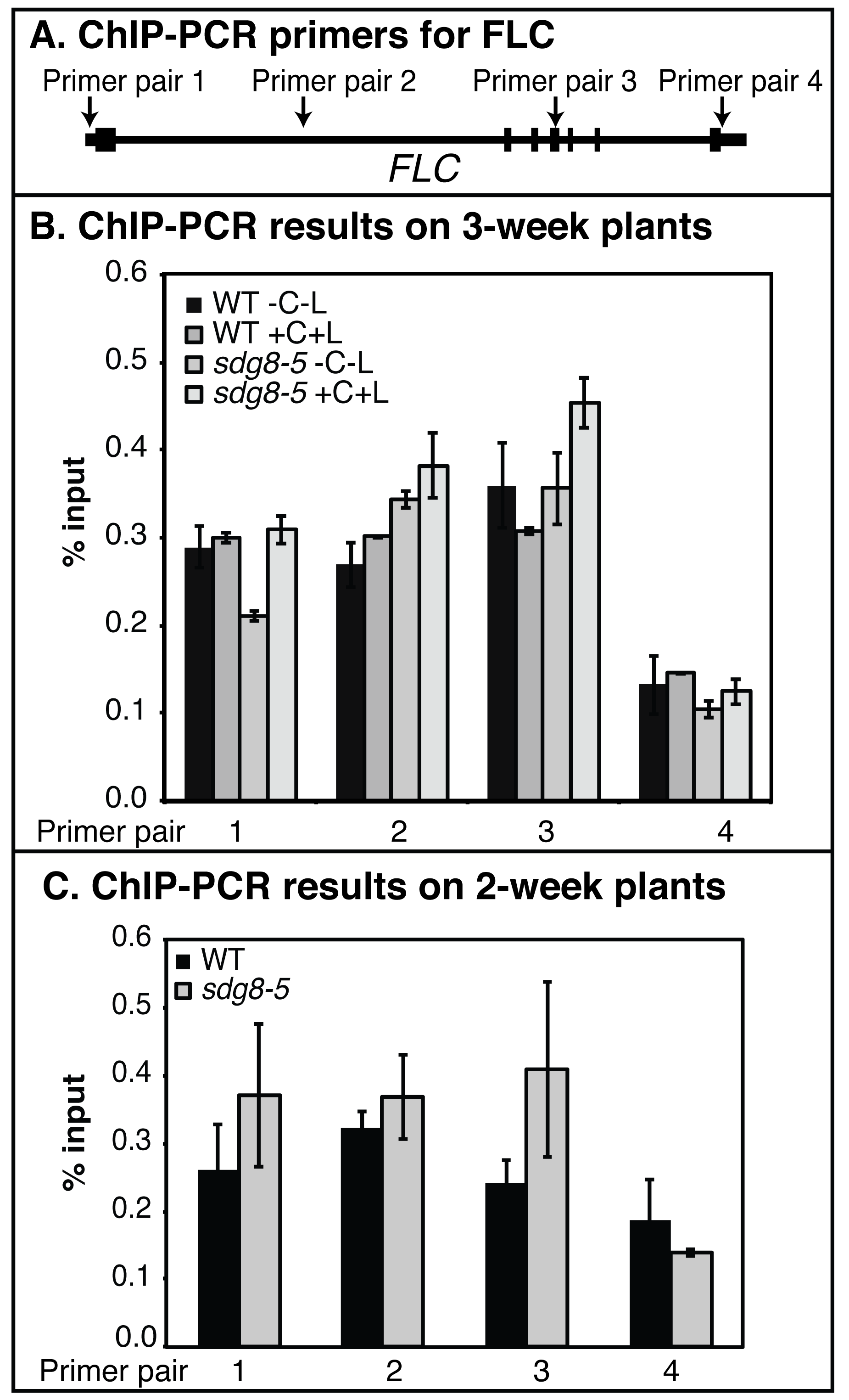
**

**Fig. S6 ChIP-PCR validation of H3K36me3 level of FLC.** ChIP-PCR was performed to validate the H3K36me3 ChIP-Seq results of *FLC* in *sdg8-5* compared to WT. (A) The ChIP-PCR primers were designed to span the genic region of *FLC*. ChIP-PCR results from 3-week-old plants (B) and 2-week-old plants (C) were shown. C: carbon; L: light.


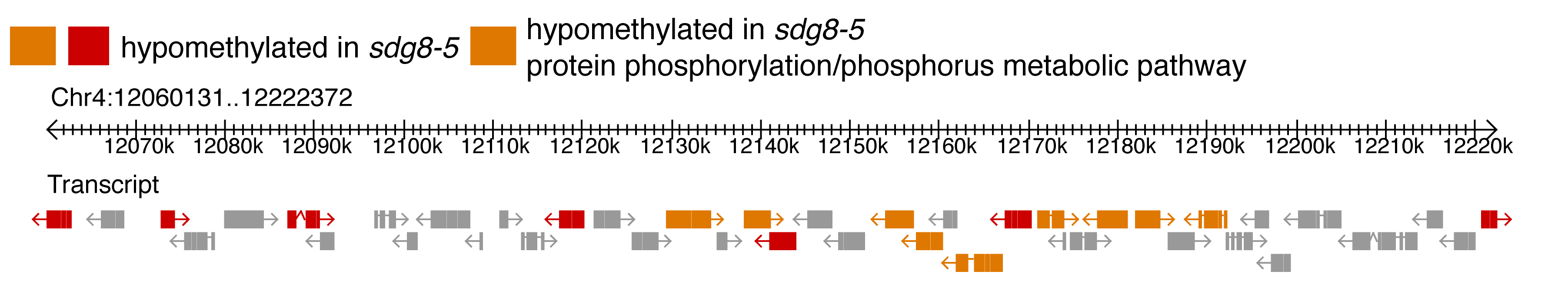
**Fig. S7**. Example of a gene cluster targeted by SDG8 for histone methylation. This gene cluster is significantly enriched (FDR<0.1) with genes in protein phosphorylation pathway.


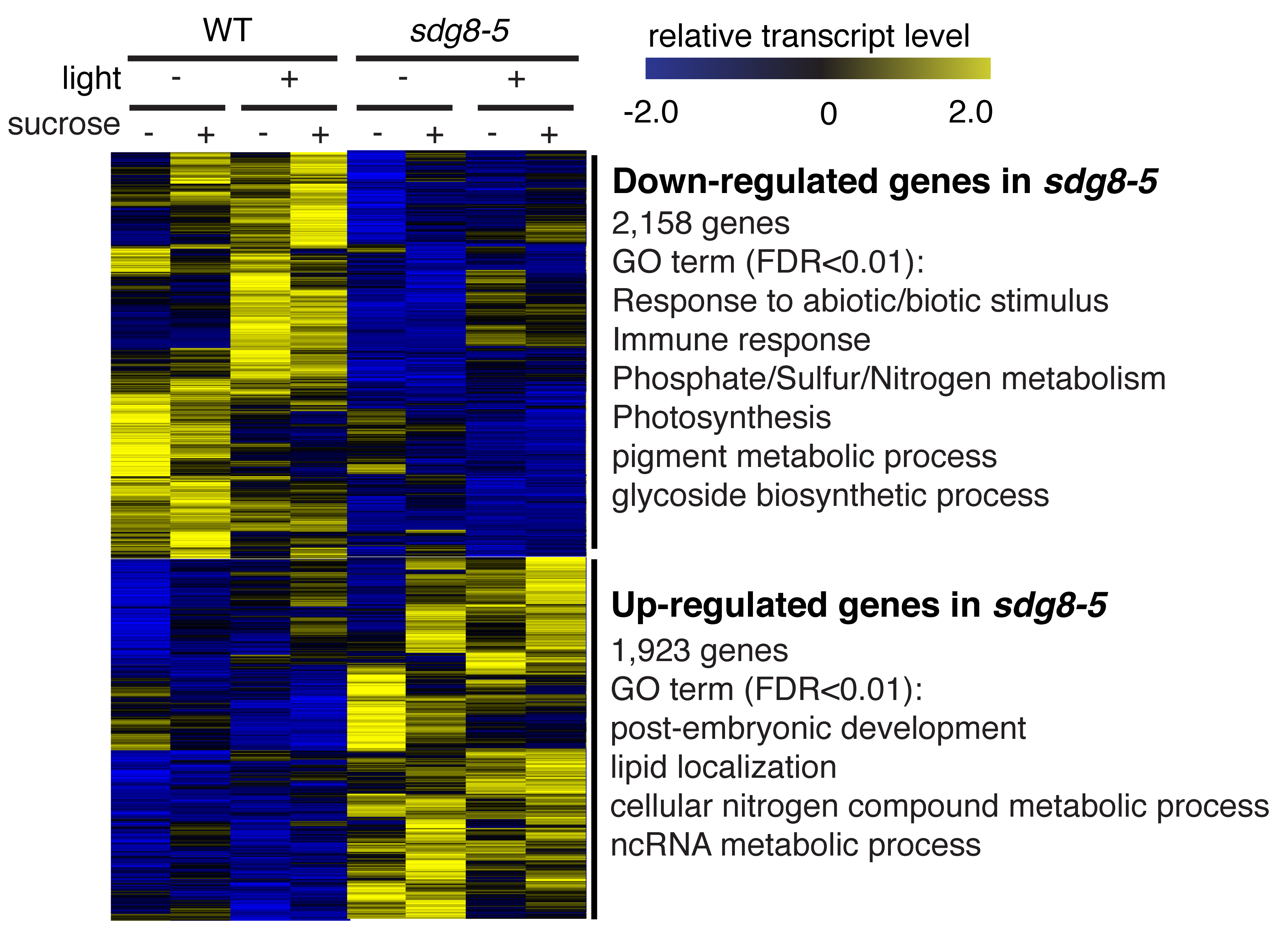


**Fig. S8** Genes misexpressed in *sdg8-5* compared to WT in transcriptomic analysis.


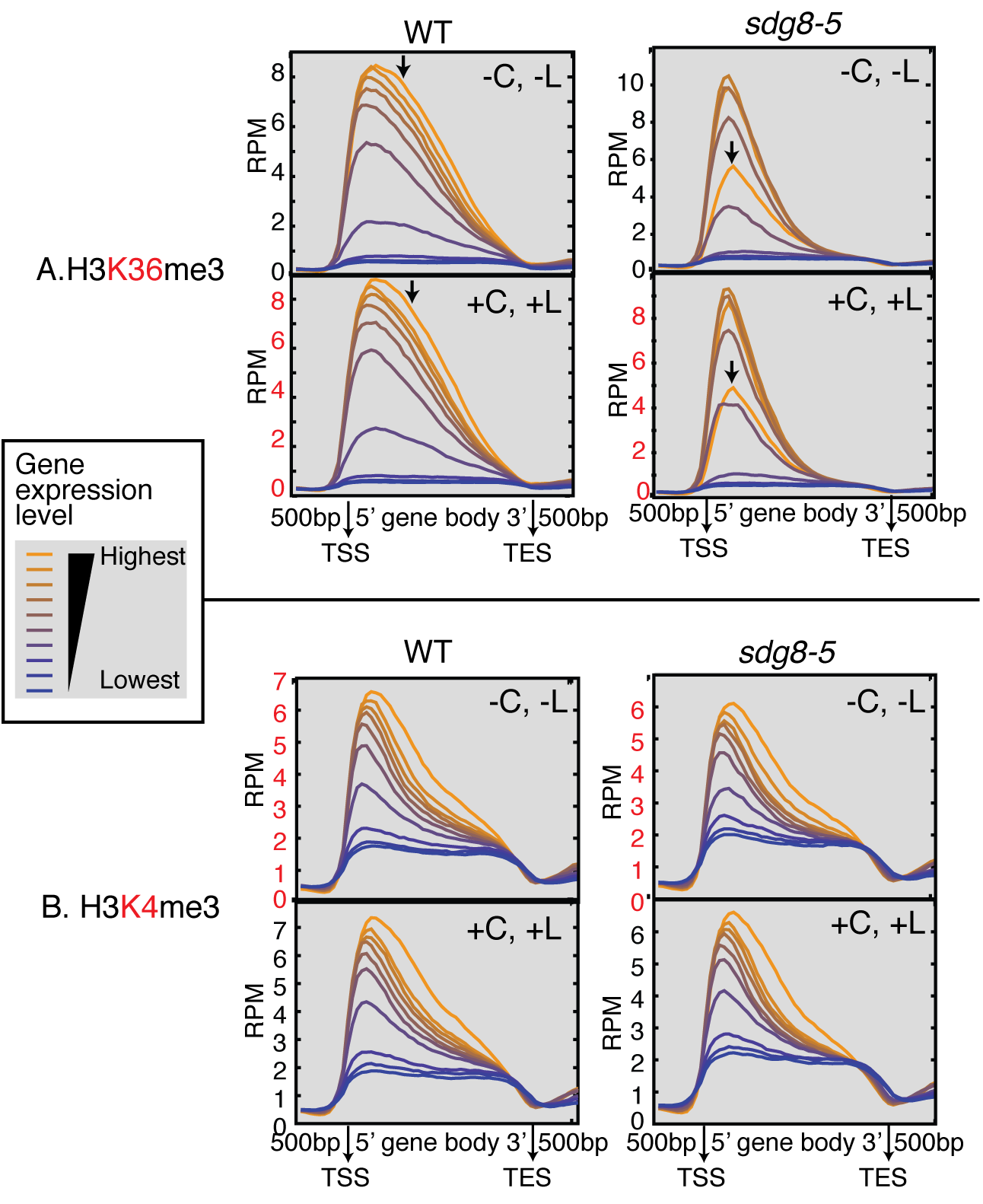


**Fig. S9. Correlation between H3K36me3 (A) or H3K4me3 (B) level and gene expression level**. All genes in the ATH1 array were ranked by their expression level from high to low, and classified into 10 expression tiers. For each tier, the median of H3K4me3 and H3K36me3 level along the gene region (500bp upstream->gene body->500bp downstream) across all genes in the expression tier was plotted. The Y axis indicates the H3K4me3 level or H3K36me3 level, measured by the number of times a region was detected by ChIP sequencing reads, normalized to the library size as reads per million (RPM)). The highest expression tier, represented by the bright orange line (highlighted with an arrow), is noticeably hypomethylated with the H3K36me3 marks in *sdg8-5* plants. TSS and TES represent the start and end of the annotated mRNA.


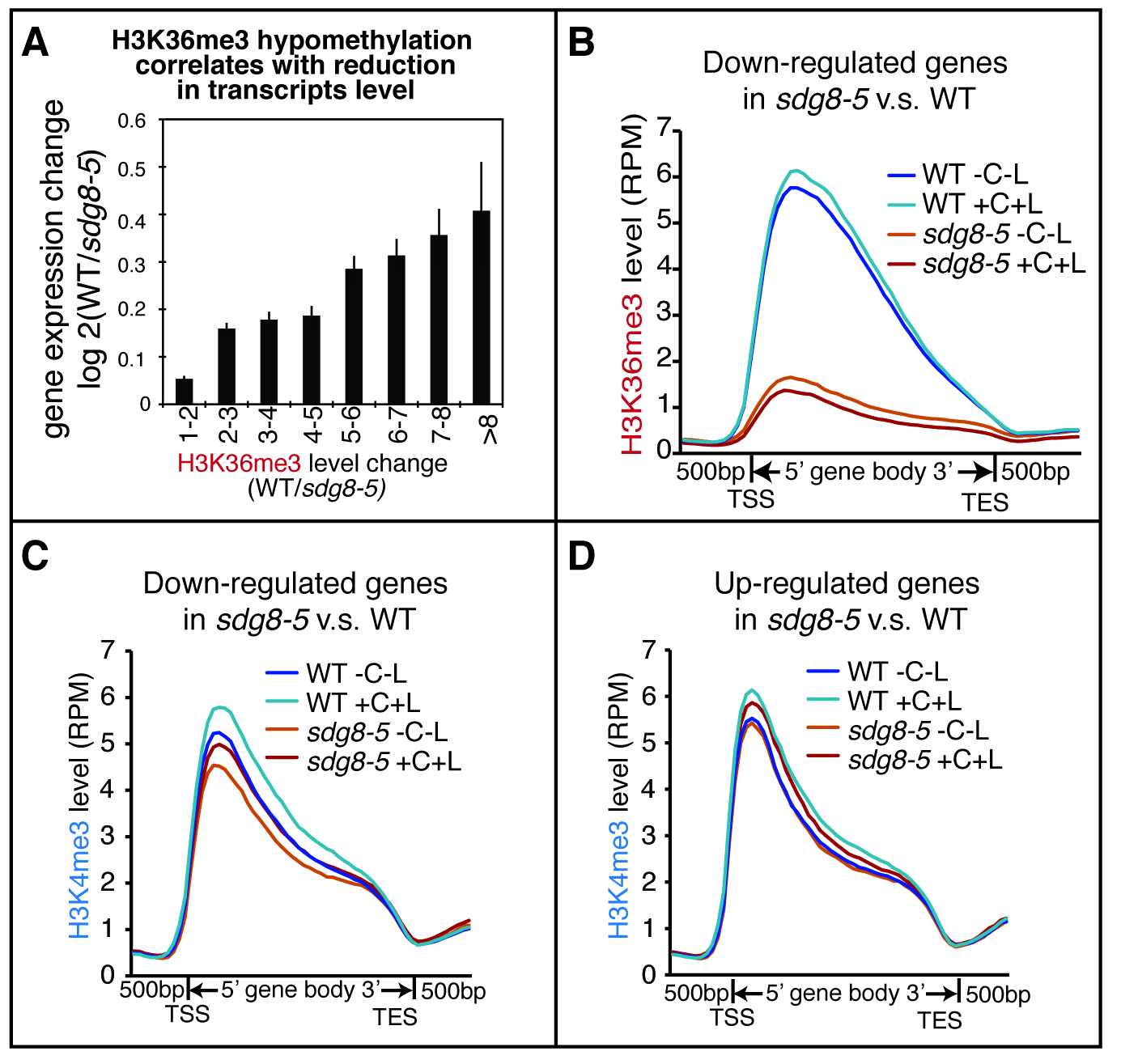


**Fig. S10.** **Integrated analysis of epigenome and transcriptome.** (A) H3K36me3 hypo-methylation in *sdg8-5* compared to WT is positively correlated with the relative reduction of gene expression. (B) Down-regulated genes in *sdg8-5* compared to WT (2158 genes) show a reduction of H3K36me3 level in *sdg8-5* compared to WT. By contrast, H3K4me3 level is comparable between *sdg8-5* and WT for the down-regulated (C) or up-regulated genes (D) in *sdg8-5* vs. WT. TSS and TES represent the start and end of the annotated mRNA.

**
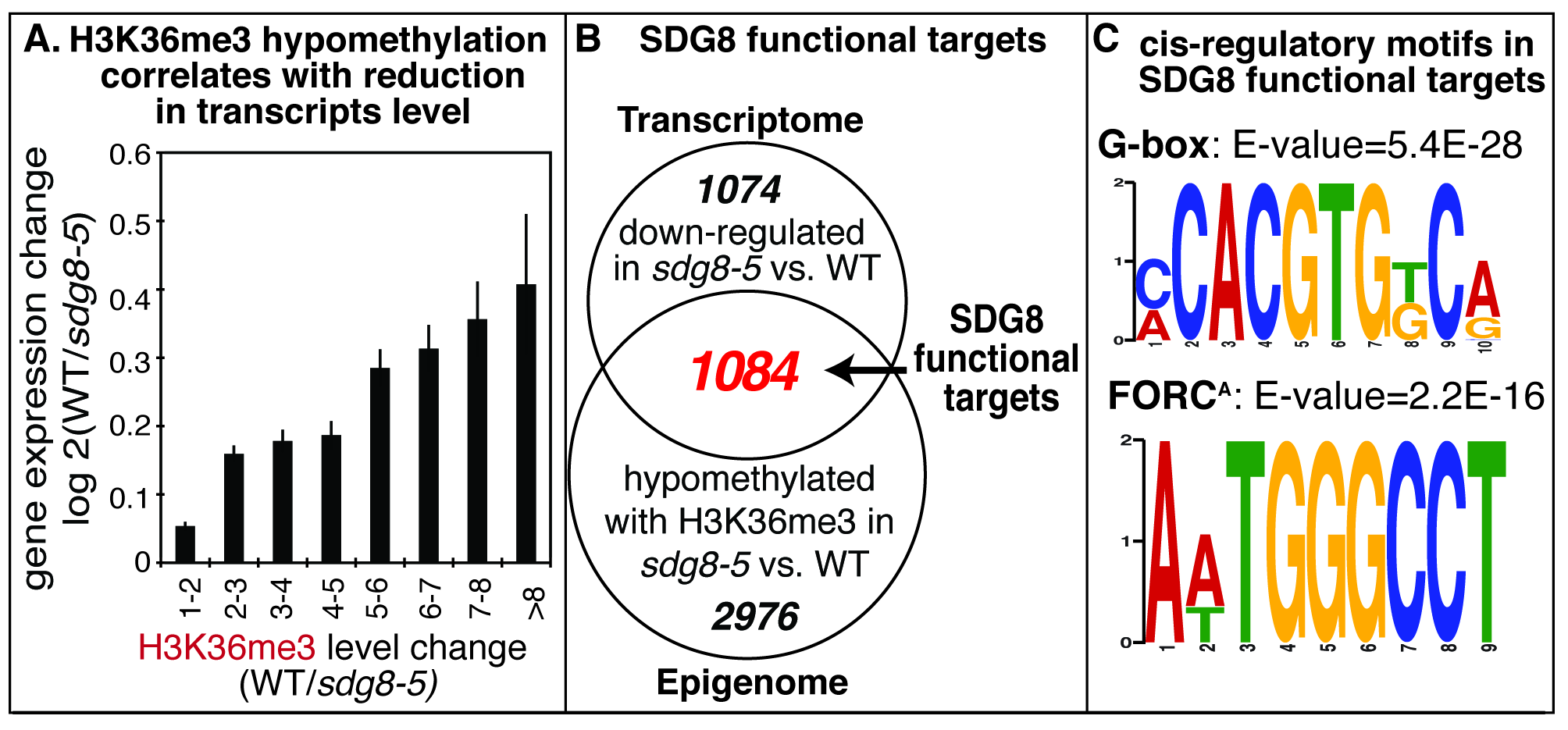
**

**Fig. S11.** Over-represented cis-regulatory motifs in the promoters of the 1084 functional target genes of SDG8. The motif analysis was performed with MEME [4].


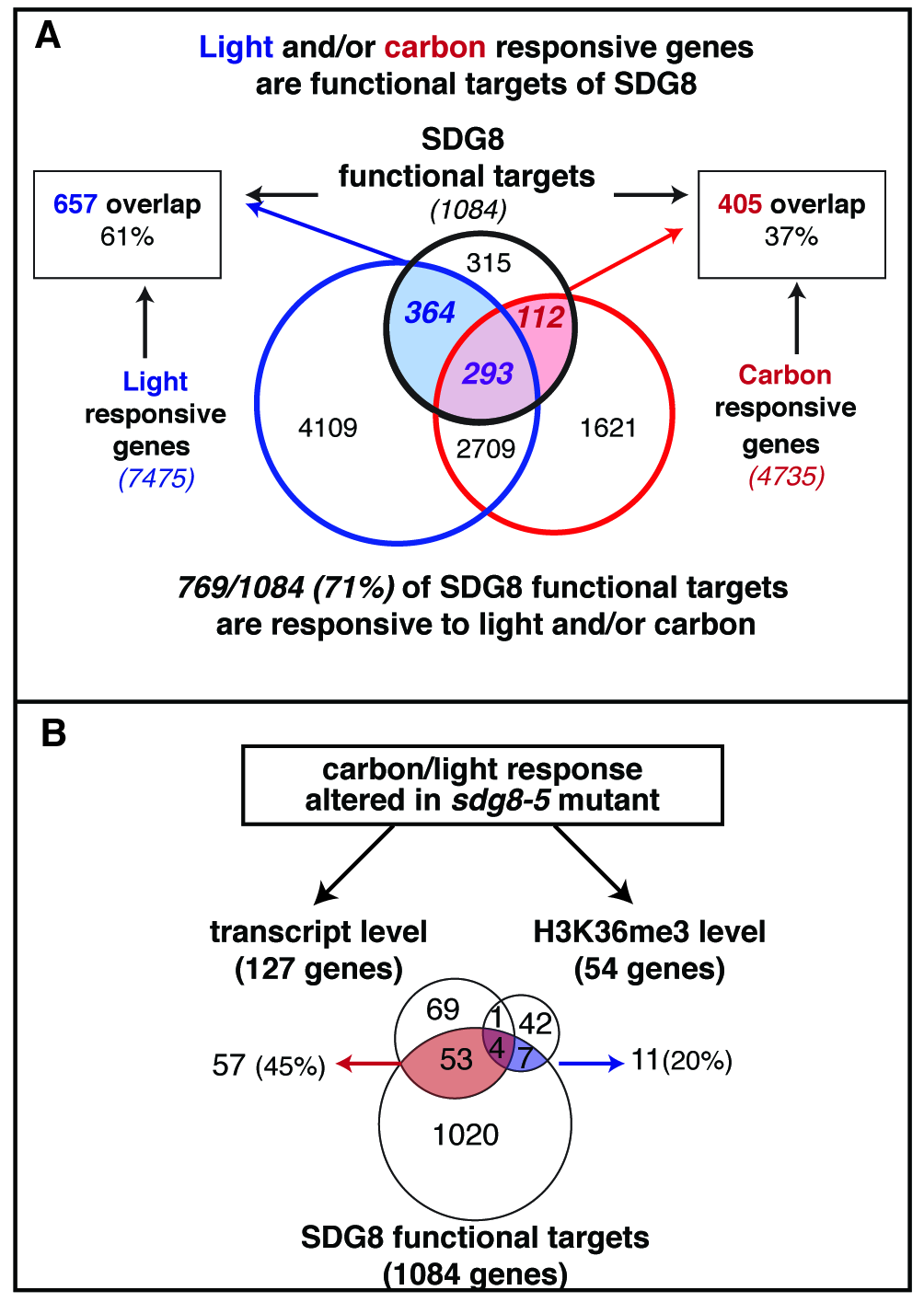


**Fig. S12.** **SDG8 functional targets are enriched in carbon and light responsive genes.** (A) Majority (71%) of the 1,084 functional targets of SDG8 (whose H3K36me3 and expression level are reduced in *sdg8-5* compared to WT) are responsive to carbon, or light, or both. (B) SDG8 functional targets whose transcriptional and/or epigenetic response to light/carbon is disrupted in *sdg8-5* deletion mutant. 1,084 functional targets of SDG8 have a significant overlap with the 127 genes significantly responsive to a genotype (G) x light (L) interaction, and 54 genes whose H3K36me3 levels are induced by carbon (C) and light (L) in WT, but not in *sdg8-5*.


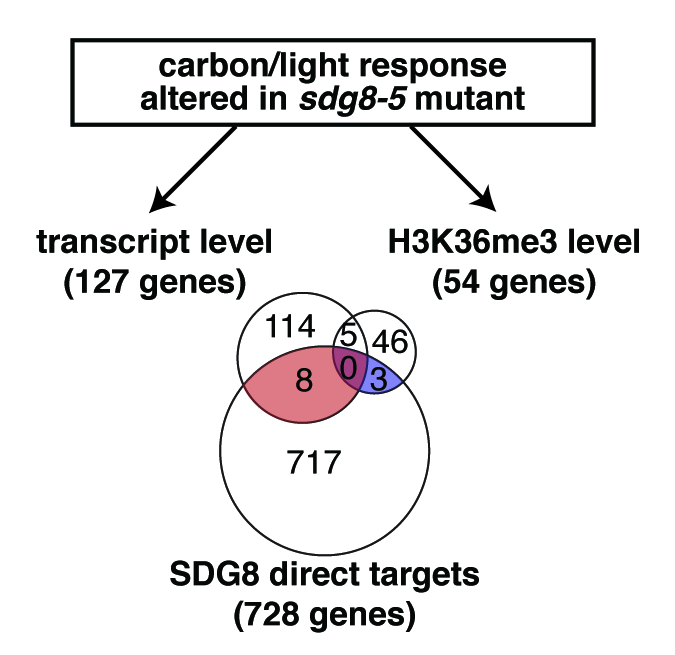


**Fig. S13.** Overlaps between SDG8 direct targets with genes whose transcriptional and/or epigenetic response to light/carbon is disrupted in *sdg8-5* deletion mutant.

**Supplemental Tables**

Table S1. Carbon (C) and light (L) regulation of ASN1 expression in WT, *cli186 (now renamed sdg8-5)* and *cli186* transformed with genomic SDG8 sequence (cli186-gSDG8), measured by qPCR in etiolated seedlings as described in [1]. The fold change compared to untreated plants is listed, while negative numbers indicate gene repression. The asterisks indicate t-test p-value<0.05.

|  | ***WT*** | ***cli186***  ***(sdg8-5)*** | ***cli186-***  ***gSDG8 (5G)*** | ***cli186-***  ***gSDG8 (10A)*** | ***cli186-***  ***gSDG8***  ***(23H)*** |
| --- | --- | --- | --- | --- | --- |
| CL | -94.8* | -2.3* | -70.5* | -115.5* | -39.8* |
| L | -6.4 | -1.6 | -7.9 | -3.5 | -3.8 |
| C | -13.6* | -1.6* | -10.6* | -28.5* | -10.4* |

Table S2. Flowering time and stage of *cli186* (now renamed *sdg8-5*) and a previously reported SDG8 mutant *fn210*. Seeds were sterilized and sown in MS agar plates (0.1% Sucrose, 2 mM KNO3). After about one week of growth in the MS plates in an Intellus environment controller [Percival Scientific, Perry, IA] (50 uEin m-2s-1, 24 hrs white light), seedlings were transferred to soil once the cotyledons emerged out. Plants were monitored everyday after the first week in soil. Number of leaves for individual plants was counted when plants started to bolt and the counting was continued until the last plant showed bolting. 30-40 plants were measured for each genotype. Experiments were done both in long-day (16h Light/8h Dark, 50 uEin m-2s-1) and short-day (8h Light/16h dark, 50 uE in m-2s-1) growth conditions. The asterisks indicate t-test p-value<1E-4.

|  | **Short Days (8hL/16hD)** | | **Long Days (16hL/8hD)** | |
| --- | --- | --- | --- | --- |
| Genotype | No. of Leaves | No. of Days | No. of Leaves | No. of Days |
| WT | 11* (+/- 0.31) | 43* (+/- 0.79) | 6 | 28* (+/- 0.48) |
| *cli186 (sdg8-5)* | 7* (+/- 0.10) | 32* (+/- 0.56) | 6 | 24* (+/- 0.66) |
| Col-0 | 11* (+/- 0.40) | 43* (+/- 0.78) | 6 | 26* (+/- 0.43) |
| *fn210* | 7* (+/- 0.17) | 30* (+/- 0.27) | 6 | 21* (+/- 0.28) |

Table S3. Carbon (C) and light (L) regulation of ASN1 expression in *cli186* *(*now renamed *sdg8-5)* and previously reported SDG8 mutant *fn210*, measured by qPCR in etiolated seedlings as described in [1]. The fold change compared to untreated plants is listed, while negative numbers indicate gene repression.

|  | **WT** | ***cli186***  ***(sdg8-5)*** | **Col-0** | ***fn210*** |
| --- | --- | --- | --- | --- |
| CL | -51.9 | -6.4 | -92.0 | -20.2 |
| L | -2.8 | -1.4 | -2.3 | -1.2 |
| C | -8.9 | -1.8 | -8.8 | -3.9 |

Table S4. Statistics of ChIP-Seq. Chr: Chromosome. pCpL: Carbon and Light treated sample; mCmL: untreated sample.

| **Library** | **Number of Raw read pairs** | **Number of Chr mapped fragments** | **%**  **Fragments mapped to Chr** | **Number of non-redundant Chr mapped fragments** | **median size of Chr mapped fragments**  **(bp)** |
| --- | --- | --- | --- | --- | --- |
| sdg8-5_mCmL_H3K36_rep1 | 40,269,866 | 34,757,412 | 86.31 | 32,444,727 | 205 |
| sdg8-5_mCmL_H3K36_rep2 | 23,321,644 | 19,705,524 | 84.49 | 19,200,718 | 160 |
| sdg8-5_mCmL_H3K4_rep1 | 35,153,794 | 30,160,219 | 85.80 | 28,101,467 | 175 |
| sdg8-5_mCmL_H3K4_rep2 | 20,520,576 | 17,947,197 | 87.46 | 17,531,578 | 161 |
| sdg8-5_mCmL_input_rep1 | 34,188,241 | 22,983,207 | 67.23 | 21,730,013 | 184 |
| sdg8-5_mCmL_input_rep2 | 27,716,455 | 16,544,869 | 59.69 | 16,194,532 | 143 |
| sdg8-5_pCpL_H3K36_rep1 | 30,600,184 | 26,113,431 | 85.34 | 24,199,198 | 184 |
| sdg8-5_pCpL_H3K36_rep2 | 25,066,031 | 21,450,291 | 85.58 | 20,970,706 | 169 |
| sdg8-5_pCpL_H3K4_rep1 | 33,418,365 | 28,211,840 | 84.42 | 24,473,200 | 185 |
| sdg8-5_pCpL_H3K4_rep2 | 21,500,200 | 18,380,131 | 85.49 | 18,061,028 | 165 |
| sdg8-5_pCpL_input_rep1 | 35,595,470 | 24,322,500 | 68.33 | 21,217,525 | 165 |
| sdg8-5_pCpL_input_rep2 | 23,637,412 | 14,158,267 | 59.90 | 13,859,590 | 149 |
| WT_mCmL_H3K36_rep1 | 33,051,288 | 29,324,994 | 88.73 | 27,734,731 | 192 |
| WT_mCmL_H3K36_rep2 | 18,235,638 | 14,631,384 | 80.24 | 14,142,472 | 141 |
| WT_mCmL_H3K4_rep1 | 36,342,207 | 31,726,224 | 87.30 | 28,845,302 | 179 |
| WT_mCmL_H3K4_rep2 | 12,071,111 | 10,790,954 | 89.39 | 10,669,444 | 174 |
| WT_mCmL_input_rep1 | 34,198,102 | 23,298,491 | 68.13 | 21,849,336 | 177 |
| WT_mCmL_input_rep2 | 19,647,905 | 12,158,491 | 61.88 | 11,851,974 | 154 |
| WT_pCpL_H3K36_rep1 | 30,380,168 | 26,857,472 | 88.40 | 23,085,690 | 191 |
| WT_pCpL_H3K36_rep2 | 24,663,493 | 21,463,945 | 87.03 | 21,067,550 | 166 |
| WT_pCpL_H3K4_rep1 | 30,727,793 | 26,775,661 | 87.14 | 23,369,181 | 195 |
| WT_pCpL_H3K4_rep2 | 16,375,496 | 14,034,267 | 85.70 | 13,835,645 | 161 |
| WT_pCpL_input_rep1 | 32,717,060 | 23,951,655 | 73.21 | 20,299,775 | 177 |
| WT_pCpL_input_rep2 | 15,318,089 | 10,387,834 | 67.81 | 10,247,129 | 154 |

Table S5. Significantly over-represented GO terms (FDR adjusted p-value <1E-6) in the 4060 genes hypomethylated with H3K36me3 in *sdg8-5* mutant compared to WT, determined using AgriGO[5].

| GO_acc | Term | FDR adjusted Pvalue |
| --- | --- | --- |
| GO:0050896 | response to stimulus | 3.00E-63 |
| GO:0006950 | response to stress | 1.60E-51 |
| GO:0006952 | defense response | 2.00E-48 |
| GO:0042221 | response to chemical stimulus | 3.30E-47 |
| GO:0010033 | response to organic substance | 2.60E-44 |
| GO:0045087 | innate immune response | 1.30E-39 |
| GO:0002376 | immune system process | 2.20E-38 |
| GO:0006955 | immune response | 2.20E-38 |
| GO:0065007 | biological regulation | 2.30E-38 |
| GO:0048583 | regulation of response to stimulus | 1.30E-37 |
| GO:0009987 | cellular process | 3.20E-37 |
| GO:0031347 | regulation of defense response | 3.30E-37 |
| GO:0050789 | regulation of biological process | 5.60E-37 |
| GO:0080134 | regulation of response to stress | 1.40E-36 |
| GO:0044237 | cellular metabolic process | 2.80E-35 |
| GO:0008152 | metabolic process | 4.00E-33 |
| GO:0050794 | regulation of cellular process | 6.40E-33 |
| GO:0051707 | response to other organism | 9.00E-33 |
| GO:0010941 | regulation of cell death | 2.30E-32 |
| GO:0034050 | host programmed cell death induced by symbiont | 1.90E-31 |
| GO:0012501 | programmed cell death | 2.70E-31 |
| GO:0043067 | regulation of programmed cell death | 3.30E-31 |
| GO:0009626 | plant-type hypersensitive response | 3.30E-31 |
| GO:0009751 | response to salicylic acid stimulus | 4.70E-31 |
| GO:0007165 | signal transduction | 4.80E-31 |
| GO:0023052 | signaling | 6.00E-31 |
| GO:0009607 | response to biotic stimulus | 8.80E-31 |
| GO:0010363 | regulation of plant-type hypersensitive response | 8.80E-31 |
| GO:0009628 | response to abiotic stimulus | 8.80E-31 |
| GO:0009719 | response to endogenous stimulus | 8.90E-31 |
| GO:0009814 | defense response, incompatible interaction | 9.50E-31 |
| GO:0080135 | regulation of cellular response to stress | 2.10E-30 |
| GO:0050776 | regulation of immune response | 2.40E-30 |
| GO:0002682 | regulation of immune system process | 2.40E-30 |
| GO:0045088 | regulation of innate immune response | 2.40E-30 |
| GO:0051716 | cellular response to stimulus | 3.70E-30 |
| GO:0009627 | systemic acquired resistance | 3.70E-30 |
| GO:0008219 | cell death | 5.60E-30 |
| GO:0016265 | death | 5.60E-30 |
| GO:0006612 | protein targeting to membrane | 1.50E-29 |
| GO:0009617 | response to bacterium | 1.70E-29 |
| GO:0070887 | cellular response to chemical stimulus | 2.80E-29 |
| GO:0009863 | salicylic acid mediated signaling pathway | 3.30E-29 |
| GO:0071446 | cellular response to salicylic acid stimulus | 5.40E-29 |
| GO:0023060 | signal transmission | 1.20E-28 |
| GO:0023046 | signaling process | 1.30E-28 |
| GO:0044283 | small molecule biosynthetic process | 7.60E-28 |
| GO:0007242 | intracellular signaling cascade | 4.10E-27 |
| GO:0009743 | response to carbohydrate stimulus | 4.80E-27 |
| GO:0048585 | negative regulation of response to stimulus | 8.50E-27 |
| GO:0031348 | negative regulation of defense response | 2.10E-26 |
| GO:0044281 | small molecule metabolic process | 2.80E-25 |
| GO:0071495 | cellular response to endogenous stimulus | 4.80E-25 |
| GO:0009058 | biosynthetic process | 1.10E-24 |
| GO:0009753 | response to jasmonic acid stimulus | 1.50E-24 |
| GO:0071310 | cellular response to organic substance | 2.00E-24 |
| GO:0033554 | cellular response to stress | 2.30E-24 |
| GO:0044238 | primary metabolic process | 2.30E-24 |
| GO:0009862 | systemic acquired resistance, salicylic acid mediated signaling pathway | 2.10E-23 |
| GO:0006725 | cellular aromatic compound metabolic process | 3.50E-23 |
| GO:0051704 | multi-organism process | 3.80E-22 |
| GO:0044249 | cellular biosynthetic process | 9.40E-22 |
| GO:0010200 | response to chitin | 1.10E-21 |
| GO:0000165 | MAPKKK cascade | 5.10E-21 |
| GO:0007243 | protein kinase cascade | 1.10E-20 |
| GO:0006796 | phosphate metabolic process | 1.30E-20 |
| GO:0006793 | phosphorus metabolic process | 1.40E-20 |
| GO:0006970 | response to osmotic stress | 4.30E-20 |
| GO:0031323 | regulation of cellular metabolic process | 4.40E-20 |
| GO:0009867 | jasmonic acid mediated signaling pathway | 9.20E-20 |
| GO:0071395 | cellular response to jasmonic acid stimulus | 9.20E-20 |
| GO:0034641 | cellular nitrogen compound metabolic process | 9.50E-20 |
| GO:0009651 | response to salt stress | 1.40E-19 |
| GO:0009725 | response to hormone stimulus | 1.90E-19 |
| GO:0046394 | carboxylic acid biosynthetic process | 4.70E-19 |
| GO:0016053 | organic acid biosynthetic process | 4.70E-19 |
| GO:0009737 | response to abscisic acid stimulus | 5.60E-19 |
| GO:0006790 | sulfur metabolic process | 5.80E-19 |
| GO:0042742 | defense response to bacterium | 1.00E-18 |
| GO:0006519 | cellular amino acid and derivative metabolic process | 1.00E-18 |
| GO:0042743 | hydrogen peroxide metabolic process | 1.10E-18 |
| GO:0043436 | oxoacid metabolic process | 2.70E-18 |
| GO:0019752 | carboxylic acid metabolic process | 2.70E-18 |
| GO:0006082 | organic acid metabolic process | 3.20E-18 |
| GO:0042180 | cellular ketone metabolic process | 7.90E-18 |
| GO:0006800 | oxygen and reactive oxygen species metabolic process | 1.10E-17 |
| GO:0019438 | aromatic compound biosynthetic process | 1.70E-17 |
| GO:0080010 | regulation of oxygen and reactive oxygen species metabolic process | 3.90E-17 |
| GO:0010310 | regulation of hydrogen peroxide metabolic process | 7.80E-17 |
| GO:0019222 | regulation of metabolic process | 1.70E-16 |
| GO:0019344 | cysteine biosynthetic process | 2.00E-16 |
| GO:0006534 | cysteine metabolic process | 4.10E-16 |
| GO:0044272 | sulfur compound biosynthetic process | 5.10E-16 |
| GO:0080090 | regulation of primary metabolic process | 6.70E-16 |
| GO:0009697 | salicylic acid biosynthetic process | 9.80E-16 |
| GO:0009696 | salicylic acid metabolic process | 1.30E-15 |
| GO:0009070 | serine family amino acid biosynthetic process | 1.30E-15 |
| GO:0006807 | nitrogen compound metabolic process | 1.50E-15 |
| GO:0043412 | macromolecule modification | 1.90E-15 |
| GO:0009266 | response to temperature stimulus | 2.80E-15 |
| GO:0006468 | protein amino acid phosphorylation | 3.60E-15 |
| GO:0060548 | negative regulation of cell death | 5.60E-15 |
| GO:0009889 | regulation of biosynthetic process | 7.00E-15 |
| GO:0031326 | regulation of cellular biosynthetic process | 8.20E-15 |
| GO:0000096 | sulfur amino acid metabolic process | 1.00E-14 |
| GO:0009069 | serine family amino acid metabolic process | 1.50E-14 |
| GO:0009620 | response to fungus | 2.30E-14 |
| GO:0016310 | phosphorylation | 2.90E-14 |
| GO:0043069 | negative regulation of programmed cell death | 3.30E-14 |
| GO:0006575 | cellular amino acid derivative metabolic process | 3.60E-14 |
| GO:0019748 | secondary metabolic process | 3.60E-14 |
| GO:0051234 | establishment of localization | 4.60E-14 |
| GO:0032870 | cellular response to hormone stimulus | 8.30E-14 |
| GO:0032268 | regulation of cellular protein metabolic process | 1.10E-13 |
| GO:0006520 | cellular amino acid metabolic process | 1.50E-13 |
| GO:0006810 | transport | 1.50E-13 |
| GO:0050832 | defense response to fungus | 1.80E-13 |
| GO:0009605 | response to external stimulus | 1.80E-13 |
| GO:0009755 | hormone-mediated signaling pathway | 2.30E-13 |
| GO:0051179 | localization | 2.70E-13 |
| GO:0044106 | cellular amine metabolic process | 2.80E-13 |
| GO:0035304 | regulation of protein amino acid dephosphorylation | 4.00E-13 |
| GO:0031399 | regulation of protein modification process | 4.00E-13 |
| GO:0009308 | amine metabolic process | 5.10E-13 |
| GO:0044260 | cellular macromolecule metabolic process | 5.30E-13 |
| GO:0006464 | protein modification process | 5.70E-13 |
| GO:0009416 | response to light stimulus | 5.70E-13 |
| GO:0035303 | regulation of dephosphorylation | 6.50E-13 |
| GO:0010035 | response to inorganic substance | 6.50E-13 |
| GO:0044271 | cellular nitrogen compound biosynthetic process | 6.70E-13 |
| GO:0051246 | regulation of protein metabolic process | 6.90E-13 |
| GO:0009415 | response to water | 1.10E-12 |
| GO:0048519 | negative regulation of biological process | 1.30E-12 |
| GO:0009414 | response to water deprivation | 1.40E-12 |
| GO:0070838 | divalent metal ion transport | 1.40E-12 |
| GO:0032787 | monocarboxylic acid metabolic process | 1.60E-12 |
| GO:0000097 | sulfur amino acid biosynthetic process | 2.20E-12 |
| GO:0006091 | generation of precursor metabolites and energy | 2.80E-12 |
| GO:0051606 | detection of stimulus | 3.80E-12 |
| GO:0009611 | response to wounding | 4.00E-12 |
| GO:0043687 | post-translational protein modification | 6.10E-12 |
| GO:0034976 | response to endoplasmic reticulum stress | 6.10E-12 |
| GO:0023034 | intracellular signaling pathway | 1.30E-11 |
| GO:0006972 | hyperosmotic response | 1.90E-11 |
| GO:0006811 | ion transport | 2.20E-11 |
| GO:0006470 | protein amino acid dephosphorylation | 2.60E-11 |
| GO:0043170 | macromolecule metabolic process | 3.50E-11 |
| GO:0046483 | heterocycle metabolic process | 7.40E-11 |
| GO:0009314 | response to radiation | 7.50E-11 |
| GO:0023033 | signaling pathway | 1.10E-10 |
| GO:0046686 | response to cadmium ion | 1.20E-10 |
| GO:0009893 | positive regulation of metabolic process | 1.30E-10 |
| GO:0016311 | dephosphorylation | 1.30E-10 |
| GO:0032879 | regulation of localization | 1.60E-10 |
| GO:0031325 | positive regulation of cellular metabolic process | 1.60E-10 |
| GO:0009963 | positive regulation of flavonoid biosynthetic process | 1.70E-10 |
| GO:0044282 | small molecule catabolic process | 2.10E-10 |
| GO:0009595 | detection of biotic stimulus | 2.20E-10 |
| GO:0006605 | protein targeting | 2.30E-10 |
| GO:0010556 | regulation of macromolecule biosynthetic process | 3.30E-10 |
| GO:0002237 | response to molecule of bacterial origin | 3.40E-10 |
| GO:0002679 | respiratory burst during defense response | 3.50E-10 |
| GO:0045730 | respiratory burst | 3.50E-10 |
| GO:0055082 | cellular chemical homeostasis | 3.70E-10 |
| GO:0016052 | carbohydrate catabolic process | 4.20E-10 |
| GO:0051171 | regulation of nitrogen compound metabolic process | 4.80E-10 |
| GO:0050801 | ion homeostasis | 5.00E-10 |
| GO:0007623 | circadian rhythm | 5.10E-10 |
| GO:0048511 | rhythmic process | 5.10E-10 |
| GO:0044275 | cellular carbohydrate catabolic process | 5.50E-10 |
| GO:0019684 | photosynthesis, light reaction | 5.70E-10 |
| GO:0051252 | regulation of RNA metabolic process | 5.90E-10 |
| GO:0006873 | cellular ion homeostasis | 6.50E-10 |
| GO:0010038 | response to metal ion | 7.40E-10 |
| GO:0009891 | positive regulation of biosynthetic process | 7.60E-10 |
| GO:0031328 | positive regulation of cellular biosynthetic process | 7.60E-10 |
| GO:0009738 | abscisic acid mediated signaling pathway | 7.80E-10 |
| GO:0043455 | regulation of secondary metabolic process | 8.10E-10 |
| GO:0042398 | cellular amino acid derivative biosynthetic process | 8.40E-10 |
| GO:0071215 | cellular response to abscisic acid stimulus | 9.60E-10 |
| GO:0019220 | regulation of phosphate metabolic process | 9.60E-10 |
| GO:0051174 | regulation of phosphorus metabolic process | 9.60E-10 |
| GO:0048878 | chemical homeostasis | 9.70E-10 |
| GO:0006355 | regulation of transcription, DNA-dependent | 1.10E-09 |
| GO:0071216 | cellular response to biotic stimulus | 1.20E-09 |
| GO:0019219 | regulation of nucleobase, nucleoside, nucleotide and nucleic acid metabolic process | 1.30E-09 |
| GO:0030003 | cellular cation homeostasis | 1.30E-09 |
| GO:0045449 | regulation of transcription | 1.40E-09 |
| GO:0006007 | glucose catabolic process | 1.60E-09 |
| GO:0046365 | monosaccharide catabolic process | 1.70E-09 |
| GO:0055080 | cation homeostasis | 1.70E-09 |
| GO:0006986 | response to unfolded protein | 1.90E-09 |
| GO:0034620 | cellular response to unfolded protein | 1.90E-09 |
| GO:0019320 | hexose catabolic process | 1.90E-09 |
| GO:0009637 | response to blue light | 2.40E-09 |
| GO:0009812 | flavonoid metabolic process | 2.60E-09 |
| GO:0030968 | endoplasmic reticulum unfolded protein response | 2.80E-09 |
| GO:0009698 | phenylpropanoid metabolic process | 3.00E-09 |
| GO:0071445 | cellular response to protein stimulus | 3.00E-09 |
| GO:0048518 | positive regulation of biological process | 3.00E-09 |
| GO:0046164 | alcohol catabolic process | 3.10E-09 |
| GO:0007030 | Golgi organization | 3.70E-09 |
| GO:0009723 | response to ethylene stimulus | 3.70E-09 |
| GO:0006984 | ER-nuclear signaling pathway | 5.30E-09 |
| GO:0042044 | fluid transport | 5.80E-09 |
| GO:0006833 | water transport | 5.80E-09 |
| GO:0010218 | response to far red light | 5.90E-09 |
| GO:0060255 | regulation of macromolecule metabolic process | 6.30E-09 |
| GO:0009962 | regulation of flavonoid biosynthetic process | 8.50E-09 |
| GO:0008104 | protein localization | 8.60E-09 |
| GO:0015979 | photosynthesis | 9.00E-09 |
| GO:0006066 | alcohol metabolic process | 9.40E-09 |
| GO:0010817 | regulation of hormone levels | 9.70E-09 |
| GO:0006812 | cation transport | 1.00E-08 |
| GO:0010155 | regulation of proton transport | 1.70E-08 |
| GO:0006096 | glycolysis | 2.00E-08 |
| GO:0016051 | carbohydrate biosynthetic process | 2.20E-08 |
| GO:0044267 | cellular protein metabolic process | 2.40E-08 |
| GO:0042402 | cellular biogenic amine catabolic process | 2.50E-08 |
| GO:0051186 | cofactor metabolic process | 2.50E-08 |
| GO:0051049 | regulation of transport | 2.70E-08 |
| GO:0048522 | positive regulation of cellular process | 2.80E-08 |
| GO:0034284 | response to monosaccharide stimulus | 4.00E-08 |
| GO:0009746 | response to hexose stimulus | 4.00E-08 |
| GO:0010468 | regulation of gene expression | 4.40E-08 |
| GO:0070727 | cellular macromolecule localization | 4.60E-08 |
| GO:0010114 | response to red light | 4.60E-08 |
| GO:0015994 | chlorophyll metabolic process | 4.90E-08 |
| GO:0042254 | ribosome biogenesis | 5.50E-08 |
| GO:0006006 | glucose metabolic process | 6.70E-08 |
| GO:0009750 | response to fructose stimulus | 8.40E-08 |
| GO:0009813 | flavonoid biosynthetic process | 9.20E-08 |
| GO:0034613 | cellular protein localization | 9.90E-08 |
| GO:0009409 | response to cold | 1.10E-07 |
| GO:0009072 | aromatic amino acid family metabolic process | 1.10E-07 |
| GO:0006886 | intracellular protein transport | 1.10E-07 |
| GO:0006979 | response to oxidative stress | 1.20E-07 |
| GO:0044248 | cellular catabolic process | 1.20E-07 |
| GO:0010207 | photosystem II assembly | 1.30E-07 |
| GO:0005975 | carbohydrate metabolic process | 1.30E-07 |
| GO:0009056 | catabolic process | 1.30E-07 |
| GO:0043269 | regulation of ion transport | 1.40E-07 |
| GO:0022613 | ribonucleoprotein complex biogenesis | 1.50E-07 |
| GO:0016070 | RNA metabolic process | 1.50E-07 |
| GO:0019318 | hexose metabolic process | 1.60E-07 |
| GO:0042440 | pigment metabolic process | 1.60E-07 |
| GO:0001510 | RNA methylation | 2.20E-07 |
| GO:0015674 | di-, tri-valent inorganic cation transport | 2.90E-07 |
| GO:0045184 | establishment of protein localization | 3.20E-07 |
| GO:0015031 | protein transport | 3.20E-07 |
| GO:0019725 | cellular homeostasis | 3.60E-07 |
| GO:0006778 | porphyrin metabolic process | 3.80E-07 |
| GO:0033013 | tetrapyrrole metabolic process | 4.40E-07 |
| GO:0009059 | macromolecule biosynthetic process | 4.70E-07 |
| GO:0009074 | aromatic amino acid family catabolic process | 4.80E-07 |
| GO:0009310 | amine catabolic process | 5.10E-07 |
| GO:0008652 | cellular amino acid biosynthetic process | 5.30E-07 |
| GO:0046777 | protein amino acid autophosphorylation | 5.30E-07 |
| GO:0009699 | phenylpropanoid biosynthetic process | 5.60E-07 |
| GO:0042219 | cellular amino acid derivative catabolic process | 5.60E-07 |
| GO:0048767 | root hair elongation | 6.60E-07 |
| GO:0042538 | hyperosmotic salinity response | 8.20E-07 |
| GO:0046700 | heterocycle catabolic process | 8.50E-07 |
| GO:0009642 | response to light intensity | 9.80E-07 |
| GO:0019538 | protein metabolic process | 9.80E-07 |

Table S6. Flowering time of *h*SDG8*, sdg8-5* and WT. For *h*SDG8, T3 transgenic lines from two independent insertion events were assayed. Seeds were sown in soil mix (soil: perlite: vermiculite = 2:1:1) and vernalized at 4C for 4 days before grown in long-day (16h Light/8h Dark, 120 uMol m-2s-1 light). Number of leaves for individual plants was counted when plants started to bolt. 10 plants were measured for each genotype.

| Genotype | No. of Leaves | No. of Days |
| --- | --- | --- |
| WT | 14 (+/-0.7) | 28.9 (+/- 0.9) |
| *sdg8-5* | 8.3 (+/-0.4) | 27.2 (+/- 0.2) |
| *h*SDG8 1-1 | 13.8 (+/-0.6) | 28.3 (+/- 0.3) |
| *h*SDG8 2-1 | 13.1 (+/-0.5) | 28.9 (+/- 0.5) |

Table S7. Significantly over-represented GO terms (FDR adjusted p-value <1E-6) in the 728 direct targets that are hypomethylated with H3K36me3 in *sdg8-5* mutant compared to WT and bound by SDG8, determined using AgriGO[5].

| GO_acc | Term | FDR |
| --- | --- | --- |
| GO:0050896 | response to stimulus | 9.30E-53 |
| GO:0006950 | response to stress | 4.70E-46 |
| GO:0009628 | response to abiotic stimulus | 1.10E-43 |
| GO:0042221 | response to chemical stimulus | 1.30E-39 |
| GO:0009987 | cellular process | 1.70E-33 |
| GO:0044237 | cellular metabolic process | 4.40E-33 |
| GO:0006970 | response to osmotic stress | 4.10E-30 |
| GO:0010033 | response to organic substance | 4.10E-30 |
| GO:0065007 | biological regulation | 6.90E-30 |
| GO:0009651 | response to salt stress | 1.80E-29 |
| GO:0044281 | small molecule metabolic process | 4.10E-29 |
| GO:0006952 | defense response | 8.80E-28 |
| GO:0044283 | small molecule biosynthetic process | 8.00E-26 |
| GO:0050789 | regulation of biological process | 1.90E-25 |
| GO:0009266 | response to temperature stimulus | 2.10E-25 |
| GO:0009058 | biosynthetic process | 2.60E-25 |
| GO:0044249 | cellular biosynthetic process | 3.30E-25 |
| GO:0044238 | primary metabolic process | 6.10E-25 |
| GO:0008152 | metabolic process | 1.50E-24 |
| GO:0009743 | response to carbohydrate stimulus | 3.10E-24 |
| GO:0051716 | cellular response to stimulus | 3.00E-23 |
| GO:0007165 | signal transduction | 9.60E-23 |
| GO:0034641 | cellular nitrogen compound metabolic process | 2.10E-22 |
| GO:0010035 | response to inorganic substance | 2.70E-22 |
| GO:0009719 | response to endogenous stimulus | 3.70E-22 |
| GO:0051707 | response to other organism | 4.50E-22 |
| GO:0070887 | cellular response to chemical stimulus | 4.80E-22 |
| GO:0010038 | response to metal ion | 6.80E-22 |
| GO:0046686 | response to cadmium ion | 2.30E-21 |
| GO:0023046 | signaling process | 6.20E-21 |
| GO:0023060 | signal transmission | 6.20E-21 |
| GO:0050794 | regulation of cellular process | 7.10E-21 |
| GO:0006091 | generation of precursor metabolites and energy | 7.20E-21 |
| GO:0044248 | cellular catabolic process | 1.00E-20 |
| GO:0009607 | response to biotic stimulus | 1.10E-20 |
| GO:0009056 | catabolic process | 3.30E-20 |
| GO:0048583 | regulation of response to stimulus | 1.40E-19 |
| GO:0044275 | cellular carbohydrate catabolic process | 3.20E-19 |
| GO:0006007 | glucose catabolic process | 3.60E-19 |
| GO:0019320 | hexose catabolic process | 3.80E-19 |
| GO:0046365 | monosaccharide catabolic process | 5.30E-19 |
| GO:0044262 | cellular carbohydrate metabolic process | 6.50E-19 |
| GO:0044282 | small molecule catabolic process | 1.10E-18 |
| GO:0023052 | signaling | 1.20E-18 |
| GO:0046164 | alcohol catabolic process | 1.30E-18 |
| GO:0006519 | cellular amino acid and derivative metabolic process | 1.80E-18 |
| GO:0016052 | carbohydrate catabolic process | 2.50E-18 |
| GO:0006006 | glucose metabolic process | 3.10E-18 |
| GO:0009617 | response to bacterium | 3.10E-18 |
| GO:0007242 | intracellular signaling cascade | 8.70E-18 |
| GO:0051179 | localization | 9.70E-18 |
| GO:0009605 | response to external stimulus | 1.00E-17 |
| GO:0051704 | multi-organism process | 1.00E-17 |
| GO:0033554 | cellular response to stress | 1.10E-17 |
| GO:0006972 | hyperosmotic response | 1.10E-17 |
| GO:0006833 | water transport | 1.10E-17 |
| GO:0042044 | fluid transport | 1.10E-17 |
| GO:0051234 | establishment of localization | 2.40E-17 |
| GO:0044271 | cellular nitrogen compound biosynthetic process | 2.60E-17 |
| GO:0044260 | cellular macromolecule metabolic process | 6.70E-17 |
| GO:0009753 | response to jasmonic acid stimulus | 6.70E-17 |
| GO:0006810 | transport | 7.20E-17 |
| GO:0009416 | response to light stimulus | 7.40E-17 |
| GO:0019318 | hexose metabolic process | 8.20E-17 |
| GO:0006096 | glycolysis | 8.50E-17 |
| GO:0046394 | carboxylic acid biosynthetic process | 8.90E-17 |
| GO:0016053 | organic acid biosynthetic process | 8.90E-17 |
| GO:0042744 | hydrogen peroxide catabolic process | 9.10E-17 |
| GO:0019344 | cysteine biosynthetic process | 1.30E-16 |
| GO:0006534 | cysteine metabolic process | 1.50E-16 |
| GO:0005975 | carbohydrate metabolic process | 1.80E-16 |
| GO:0070301 | cellular response to hydrogen peroxide | 1.90E-16 |
| GO:0044272 | sulfur compound biosynthetic process | 1.90E-16 |
| GO:0071495 | cellular response to endogenous stimulus | 2.20E-16 |
| GO:0034050 | host programmed cell death induced by symbiont | 2.60E-16 |
| GO:0006790 | sulfur metabolic process | 2.60E-16 |
| GO:0009611 | response to wounding | 2.80E-16 |
| GO:0006066 | alcohol metabolic process | 2.90E-16 |
| GO:0007030 | Golgi organization | 3.50E-16 |
| GO:0032879 | regulation of localization | 4.10E-16 |
| GO:0009070 | serine family amino acid biosynthetic process | 5.40E-16 |
| GO:0012501 | programmed cell death | 6.60E-16 |
| GO:0005996 | monosaccharide metabolic process | 7.00E-16 |
| GO:0009626 | plant-type hypersensitive response | 9.50E-16 |
| GO:0045087 | innate immune response | 9.60E-16 |
| GO:0006807 | nitrogen compound metabolic process | 1.00E-15 |
| GO:0010941 | regulation of cell death | 1.10E-15 |
| GO:0009725 | response to hormone stimulus | 1.20E-15 |
| GO:0042742 | defense response to bacterium | 1.30E-15 |
| GO:0008219 | cell death | 1.30E-15 |
| GO:0016265 | death | 1.30E-15 |
| GO:0010363 | regulation of plant-type hypersensitive response | 1.40E-15 |
| GO:0002376 | immune system process | 1.50E-15 |
| GO:0006955 | immune response | 1.50E-15 |
| GO:0009314 | response to radiation | 1.60E-15 |
| GO:0006612 | protein targeting to membrane | 2.00E-15 |
| GO:0034599 | cellular response to oxidative stress | 2.30E-15 |
| GO:0042180 | cellular ketone metabolic process | 2.40E-15 |
| GO:0034614 | cellular response to reactive oxygen species | 2.40E-15 |
| GO:0042743 | hydrogen peroxide metabolic process | 2.40E-15 |
| GO:0031347 | regulation of defense response | 2.50E-15 |
| GO:0080135 | regulation of cellular response to stress | 2.50E-15 |
| GO:0043067 | regulation of programmed cell death | 2.70E-15 |
| GO:0043436 | oxoacid metabolic process | 2.70E-15 |
| GO:0019752 | carboxylic acid metabolic process | 2.70E-15 |
| GO:0006082 | organic acid metabolic process | 2.80E-15 |
| GO:0043170 | macromolecule metabolic process | 3.60E-15 |
| GO:0009069 | serine family amino acid metabolic process | 3.90E-15 |
| GO:0009737 | response to abscisic acid stimulus | 4.90E-15 |
| GO:0006800 | oxygen and reactive oxygen species metabolic process | 7.30E-15 |
| GO:0000097 | sulfur amino acid biosynthetic process | 7.30E-15 |
| GO:0080134 | regulation of response to stress | 7.30E-15 |
| GO:0019748 | secondary metabolic process | 9.40E-15 |
| GO:0000096 | sulfur amino acid metabolic process | 2.50E-14 |
| GO:0006979 | response to oxidative stress | 2.80E-14 |
| GO:0045088 | regulation of innate immune response | 3.30E-14 |
| GO:0044265 | cellular macromolecule catabolic process | 3.90E-14 |
| GO:0050776 | regulation of immune response | 4.40E-14 |
| GO:0002682 | regulation of immune system process | 4.40E-14 |
| GO:0015979 | photosynthesis | 5.50E-14 |
| GO:0009814 | defense response, incompatible interaction | 5.60E-14 |
| GO:0009620 | response to fungus | 5.70E-14 |
| GO:0010200 | response to chitin | 6.40E-14 |
| GO:0009409 | response to cold | 7.80E-14 |
| GO:0009057 | macromolecule catabolic process | 8.10E-14 |
| GO:0042254 | ribosome biogenesis | 1.60E-13 |
| GO:0071310 | cellular response to organic substance | 1.60E-13 |
| GO:0008104 | protein localization | 1.60E-13 |
| GO:0010155 | regulation of proton transport | 1.90E-13 |
| GO:0048585 | negative regulation of response to stimulus | 2.60E-13 |
| GO:0000302 | response to reactive oxygen species | 2.70E-13 |
| GO:0006725 | cellular aromatic compound metabolic process | 2.80E-13 |
| GO:0022613 | ribonucleoprotein complex biogenesis | 3.10E-13 |
| GO:0044106 | cellular amine metabolic process | 3.40E-13 |
| GO:0000165 | MAPKKK cascade | 4.60E-13 |
| GO:0009308 | amine metabolic process | 7.00E-13 |
| GO:0009415 | response to water | 7.20E-13 |
| GO:0009637 | response to blue light | 1.10E-12 |
| GO:0008652 | cellular amino acid biosynthetic process | 1.40E-12 |
| GO:0032870 | cellular response to hormone stimulus | 1.50E-12 |
| GO:0009414 | response to water deprivation | 1.60E-12 |
| GO:0051641 | cellular localization | 1.60E-12 |
| GO:0009755 | hormone-mediated signaling pathway | 1.60E-12 |
| GO:0009309 | amine biosynthetic process | 1.70E-12 |
| GO:0007243 | protein kinase cascade | 2.00E-12 |
| GO:0016051 | carbohydrate biosynthetic process | 2.20E-12 |
| GO:0006605 | protein targeting | 2.60E-12 |
| GO:0051649 | establishment of localization in cell | 2.90E-12 |
| GO:0006520 | cellular amino acid metabolic process | 3.30E-12 |
| GO:0042542 | response to hydrogen peroxide | 3.60E-12 |
| GO:0019684 | photosynthesis, light reaction | 4.10E-12 |
| GO:0009751 | response to salicylic acid stimulus | 6.00E-12 |
| GO:0046483 | heterocycle metabolic process | 6.20E-12 |
| GO:0050832 | defense response to fungus | 6.60E-12 |
| GO:0046907 | intracellular transport | 1.60E-11 |
| GO:0051049 | regulation of transport | 2.30E-11 |
| GO:0019438 | aromatic compound biosynthetic process | 2.30E-11 |
| GO:0009862 | systemic acquired resistance, salicylic acid mediated signaling pathway | 2.90E-11 |
| GO:0033036 | macromolecule localization | 3.00E-11 |
| GO:0043269 | regulation of ion transport | 3.40E-11 |
| GO:0044267 | cellular protein metabolic process | 3.90E-11 |
| GO:0006575 | cellular amino acid derivative metabolic process | 7.50E-11 |
| GO:0034637 | cellular carbohydrate biosynthetic process | 8.50E-11 |
| GO:0009867 | jasmonic acid mediated signaling pathway | 8.80E-11 |
| GO:0071395 | cellular response to jasmonic acid stimulus | 8.80E-11 |
| GO:0048518 | positive regulation of biological process | 8.90E-11 |
| GO:0048878 | chemical homeostasis | 8.90E-11 |
| GO:0032787 | monocarboxylic acid metabolic process | 1.10E-10 |
| GO:0070727 | cellular macromolecule localization | 1.10E-10 |
| GO:0010114 | response to red light | 1.30E-10 |
| GO:0006812 | cation transport | 1.70E-10 |
| GO:0031348 | negative regulation of defense response | 1.90E-10 |
| GO:0009627 | systemic acquired resistance | 1.90E-10 |
| GO:0015992 | proton transport | 2.10E-10 |
| GO:0006818 | hydrogen transport | 2.10E-10 |
| GO:0050801 | ion homeostasis | 2.70E-10 |
| GO:0006886 | intracellular protein transport | 2.70E-10 |
| GO:0070838 | divalent metal ion transport | 2.70E-10 |
| GO:0006811 | ion transport | 3.00E-10 |
| GO:0019538 | protein metabolic process | 4.60E-10 |
| GO:0045184 | establishment of protein localization | 4.60E-10 |
| GO:0034613 | cellular protein localization | 4.60E-10 |
| GO:0015031 | protein transport | 4.60E-10 |
| GO:0051186 | cofactor metabolic process | 6.30E-10 |
| GO:0015994 | chlorophyll metabolic process | 6.40E-10 |
| GO:0048522 | positive regulation of cellular process | 6.40E-10 |
| GO:0080090 | regulation of primary metabolic process | 6.40E-10 |
| GO:0009059 | macromolecule biosynthetic process | 7.20E-10 |
| GO:0010467 | gene expression | 7.70E-10 |
| GO:0009863 | salicylic acid mediated signaling pathway | 8.30E-10 |
| GO:0071446 | cellular response to salicylic acid stimulus | 8.80E-10 |
| GO:0032268 | regulation of cellular protein metabolic process | 9.10E-10 |
| GO:0031325 | positive regulation of cellular metabolic process | 9.60E-10 |
| GO:0034645 | cellular macromolecule biosynthetic process | 1.20E-09 |
| GO:0009893 | positive regulation of metabolic process | 1.40E-09 |
| GO:0009891 | positive regulation of biosynthetic process | 1.40E-09 |
| GO:0031328 | positive regulation of cellular biosynthetic process | 1.40E-09 |
| GO:0019222 | regulation of metabolic process | 1.70E-09 |
| GO:0010218 | response to far red light | 2.10E-09 |
| GO:0001510 | RNA methylation | 2.20E-09 |
| GO:0009642 | response to light intensity | 2.60E-09 |
| GO:0055080 | cation homeostasis | 2.80E-09 |
| GO:0031323 | regulation of cellular metabolic process | 2.90E-09 |
| GO:0006778 | porphyrin metabolic process | 3.90E-09 |
| GO:0009698 | phenylpropanoid metabolic process | 4.20E-09 |
| GO:0033013 | tetrapyrrole metabolic process | 4.20E-09 |
| GO:0015672 | monovalent inorganic cation transport | 4.40E-09 |
| GO:0009963 | positive regulation of flavonoid biosynthetic process | 4.70E-09 |
| GO:0051246 | regulation of protein metabolic process | 5.00E-09 |
| GO:0006412 | translation | 5.80E-09 |
| GO:0071215 | cellular response to abscisic acid stimulus | 5.90E-09 |
| GO:0051606 | detection of stimulus | 7.30E-09 |
| GO:0009738 | abscisic acid mediated signaling pathway | 8.10E-09 |
| GO:0043069 | negative regulation of programmed cell death | 1.20E-08 |
| GO:0060548 | negative regulation of cell death | 1.50E-08 |
| GO:0043455 | regulation of secondary metabolic process | 1.50E-08 |
| GO:0042398 | cellular amino acid derivative biosynthetic process | 1.80E-08 |
| GO:0009812 | flavonoid metabolic process | 2.40E-08 |
| GO:0032880 | regulation of protein localization | 2.40E-08 |
| GO:0006873 | cellular ion homeostasis | 3.40E-08 |
| GO:0046165 | alcohol biosynthetic process | 3.40E-08 |
| GO:0055082 | cellular chemical homeostasis | 3.60E-08 |
| GO:0031407 | oxylipin metabolic process | 3.80E-08 |
| GO:0035304 | regulation of protein amino acid dephosphorylation | 4.20E-08 |
| GO:0009889 | regulation of biosynthetic process | 5.00E-08 |
| GO:0035303 | regulation of dephosphorylation | 5.10E-08 |
| GO:0009962 | regulation of flavonoid biosynthetic process | 5.80E-08 |
| GO:0031408 | oxylipin biosynthetic process | 6.90E-08 |
| GO:0031326 | regulation of cellular biosynthetic process | 8.60E-08 |
| GO:0042592 | homeostatic process | 9.10E-08 |
| GO:0009750 | response to fructose stimulus | 9.20E-08 |
| GO:0031399 | regulation of protein modification process | 1.10E-07 |
| GO:0009723 | response to ethylene stimulus | 1.20E-07 |
| GO:0048519 | negative regulation of biological process | 1.40E-07 |
| GO:0009765 | photosynthesis, light harvesting | 1.70E-07 |
| GO:0009644 | response to high light intensity | 1.70E-07 |
| GO:0048767 | root hair elongation | 1.80E-07 |
| GO:0030003 | cellular cation homeostasis | 2.00E-07 |
| GO:0034284 | response to monosaccharide stimulus | 2.20E-07 |
| GO:0009746 | response to hexose stimulus | 2.20E-07 |
| GO:0009813 | flavonoid biosynthetic process | 2.40E-07 |
| GO:0044085 | cellular component biogenesis | 2.60E-07 |
| GO:0009699 | phenylpropanoid biosynthetic process | 2.70E-07 |
| GO:0046700 | heterocycle catabolic process | 3.10E-07 |
| GO:0046777 | protein amino acid autophosphorylation | 3.20E-07 |
| GO:0016311 | dephosphorylation | 3.20E-07 |
| GO:0009694 | jasmonic acid metabolic process | 3.60E-07 |
| GO:0010207 | photosystem II assembly | 3.80E-07 |
| GO:0043412 | macromolecule modification | 4.10E-07 |
| GO:0006470 | protein amino acid dephosphorylation | 5.10E-07 |
| GO:0023033 | signaling pathway | 5.40E-07 |
| GO:0009639 | response to red or far red light | 5.70E-07 |
| GO:0016070 | RNA metabolic process | 5.80E-07 |
| GO:0006139 | nucleobase, nucleoside, nucleotide and nucleic acid metabolic process | 6.80E-07 |
| GO:0009695 | jasmonic acid biosynthetic process | 8.20E-07 |
| GO:0000160 | two-component signal transduction system (phosphorelay) | 9.60E-07 |

Table S8. Significantly over-represented GO terms (FDR adjusted p-value <0.01) in the genes mis-expressed in *sdg8-5* mutant compared to WT, determined by AgriGO [5].

| i) Down-regulated genes in *sdg8-5* compared to WT | | |
| --- | --- | --- |
|  | Term | p-value (FDR adjusted) |
| GO:0050896 | response to stimulus | 4.80E-06 |
| GO:0045087 | innate immune response | 4.80E-06 |
| GO:0009605 | response to external stimulus | 4.80E-06 |
| GO:0002376 | immune system process | 4.80E-06 |
| GO:0006950 | response to stress | 4.80E-06 |
| GO:0006955 | immune response | 4.80E-06 |
| GO:0016144 | S-glycoside biosynthetic process | 4.80E-06 |
| GO:0019758 | glycosinolate biosynthetic process | 4.80E-06 |
| GO:0019761 | glucosinolate biosynthetic process | 4.80E-06 |
| GO:0006790 | sulfur metabolic process | 4.90E-06 |
| GO:0016143 | S-glycoside metabolic process | 1.20E-05 |
| GO:0019757 | glycosinolate metabolic process | 1.20E-05 |
| GO:0019760 | glucosinolate metabolic process | 1.20E-05 |
| GO:0006952 | defense response | 6.70E-05 |
| GO:0009416 | response to light stimulus | 6.90E-05 |
| GO:0016138 | glycoside biosynthetic process | 0.0001 |
| GO:0009314 | response to radiation | 0.00015 |
| GO:0044272 | sulfur compound biosynthetic process | 0.00015 |
| GO:0008219 | cell death | 0.00022 |
| GO:0016265 | death | 0.00022 |
| GO:0016108 | tetraterpenoid metabolic process | 0.00024 |
| GO:0016116 | carotenoid metabolic process | 0.00024 |
| GO:0051707 | response to other organism | 0.00033 |
| GO:0016137 | glycoside metabolic process | 0.0004 |
| GO:0016109 | tetraterpenoid biosynthetic process | 0.00044 |
| GO:0016117 | carotenoid biosynthetic process | 0.00044 |
| GO:0009814 | defense response, incompatible interaction | 0.00054 |
| GO:0019748 | secondary metabolic process | 0.0006 |
| GO:0012501 | programmed cell death | 0.00072 |
| GO:0009637 | response to blue light | 0.00073 |
| GO:0009607 | response to biotic stimulus | 0.00082 |
| GO:0006915 | apoptosis | 0.0009 |
| GO:0034641 | cellular nitrogen compound metabolic process | 0.001 |
| GO:0009628 | response to abiotic stimulus | 0.0021 |
| GO:0055082 | cellular chemical homeostasis | 0.0026 |
| GO:0042440 | pigment metabolic process | 0.0026 |
| GO:0016114 | terpenoid biosynthetic process | 0.0028 |
| GO:0015979 | photosynthesis | 0.0028 |
| GO:0010876 | lipid localization | 0.0028 |
| GO:0006875 | cellular metal ion homeostasis | 0.0035 |
| GO:0055065 | metal ion homeostasis | 0.0035 |
| GO:0042126 | nitrate metabolic process | 0.0038 |
| GO:0042128 | nitrate assimilation | 0.0038 |
| GO:0000103 | sulfate assimilation | 0.0045 |
| GO:0051704 | multi-organism process | 0.0046 |
| GO:0006721 | terpenoid metabolic process | 0.0048 |
| GO:0006873 | cellular ion homeostasis | 0.0052 |
| GO:0009611 | response to wounding | 0.0063 |
| GO:0006787 | porphyrin catabolic process | 0.0067 |
| GO:0033015 | tetrapyrrole catabolic process | 0.0067 |
| GO:0031668 | cellular response to extracellular stimulus | 0.0081 |
| ii) Up-regulated genes in *sdg8-5* compared to WT | | |
|  | Term | p-value (FDR adjusted) |
| GO:0009791 | post-embryonic development | 4.00E-07 |
| GO:0010876 | lipid localization | 0.00016 |
| GO:0034641 | cellular nitrogen compound metabolic process | 0.00017 |
| GO:0034660 | ncRNA metabolic process | 0.008 |

Table S9. Genes whose expression level is significantly regulated by Genotype X Light interaction (FDR<0.15 of G X L term in ANOVA).

| At2g29090 | CYP707A2, cytochrome P450, family 707, subfamily A, polypeptide 2 |
| --- | --- |
| At5g02200 | FHL, far-red-elongated hypocotyl1-like |
| At3g21670 | Major facilitator superfamily protein |
| At5g66610 | DAR7, DA1-related protein 7 |
| At1g03600 | PSB27, photosystem II family protein |
| At5g22310 | unknown protein; |
| At5g11420 | Protein of unknown function, DUF642 |
| At2g19660 | Cysteine/Histidine-rich C1 domain family protein |
| At3g19800 | Protein of unknown function (DUF177) |
| At2g04039 | unknown protein; |
| At4g19390 | Uncharacterised protein family (UPF0114) |
| At1g28600 | GDSL-like Lipase/Acylhydrolase superfamily protein |
| At1g56600 | AtGolS2, GolS2, galactinol synthase 2 |
| At3g01060 | unknown protein; Has 640 Blast hits to 638 proteins in 201 species: Archae - 0; Bacteria - 293; Metazoa - 0; Fungi - 71; Plants - 72; Viruses - 0; Other Eukaryotes - 204 (source: NCBI BLink). |
| At4g35600 | CONNEXIN 32, Protein kinase superfamily protein |
| At3g26320 | CYP71B36, cytochrome P450, family 71, subfamily B, polypeptide 36 |
| At5g19850 | alpha/beta-Hydrolases superfamily protein |
| At5g23405 | HMG-box (high mobility group) DNA-binding family protein |
| At1g74670 | Gibberellin-regulated family protein |
| At5g04160 | Nucleotide-sugar transporter family protein |
| At2g29400 | PP1-AT, TOPP1, type one protein phosphatase 1 |
| At5g63980 | ALX8, ATSAL1, FRY1, HOS2, RON1, SAL1, Inositol monophosphatase family protein |
| At3g10840 | alpha/beta-Hydrolases superfamily protein |
| At2g39450 | ATMTP11, MTP11, Cation efflux family protein |
| At4g16780 | ATHB-2, ATHB2, HAT4, HB-2, homeobox protein 2 |
| At2g47270 | sequence-specific DNA binding transcription factors;transcription regulators |
| At5g53360 | TRAF-like superfamily protein |
| At1g74440 | Protein of unknown function (DUF962) |
| At1g65370 | TRAF-like family protein |
| At4g13050 | Acyl-ACP thioesterase |
| At5g50100 | Putative thiol-disulphide oxidoreductase DCC |
| At3g16800 | Protein phosphatase 2C family protein |
| At3g57470 | Insulinase (Peptidase family M16) family protein |
| At3g16360 | AHP4, HPT phosphotransmitter 4 |
| At2g23840 | HNH endonuclease |
| At3g21890 | B-box type zinc finger family protein |
| At3g47160 | RING/U-box superfamily protein |
| At5g48900 | Pectin lyase-like superfamily protein |
| At5g56500 | TCP-1/cpn60 chaperonin family protein |
| At5g44870 | Disease resistance protein (TIR-NBS-LRR class) family |
| At5g61880 | Protein Transporter, Pam16 |
| At1g70820 | phosphoglucomutase, putative / glucose phosphomutase, putative |
| At5g02180 | Transmembrane amino acid transporter family protein |
| At2g37240 | Thioredoxin superfamily protein |
| At4g39800 | ATIPS1, ATMIPS1, MI-1-P SYNTHASE, MIPS1, myo-inositol-1-phosphate synthase 1 |
| At5g53980 | ATHB52, HB52, homeobox protein 52 |
| At1g71030 | ATMYBL2, MYBL2, MYB-like 2 |
| At4g37220 | Cold acclimation protein WCOR413 family |
| At5g57040 | Lactoylglutathione lyase / glyoxalase I family protein |
| At4g32980 | ATH1, homeobox gene 1 |
| At2g45990 | unknown protein; |
| At4g03950 | Nucleotide/sugar transporter family protein |
| At4g39640 | GGT1, gamma-glutamyl transpeptidase 1 |
| At1g07010 | Calcineurin-like metallo-phosphoesterase superfamily protein |
| At3g57480 | zinc finger (C2H2 type, AN1-like) family protein |
| At5g52910 | ATIM, timeless family protein |
| At1g13080 | CYP71B2, cytochrome P450, family 71, subfamily B, polypeptide 2 |
| At3g15760 | unknown protein; |
| At2g28200 | C2H2-type zinc finger family protein |
| At3g12610 | DRT100, Leucine-rich repeat (LRR) family protein |
| At5g37770 | CML24, TCH2, EF hand calcium-binding protein family |
| At1g66070 | Translation initiation factor eIF3 subunit |
| At4g25260 | Plant invertase/pectin methylesterase inhibitor superfamily protein |
| At4g19860 | alpha/beta-Hydrolases superfamily protein |
| At5g59070 | UDP-Glycosyltransferase superfamily protein |
| At3g53340 | NF-YB10, nuclear factor Y, subunit B10 |
| At1g52190 | Major facilitator superfamily protein |
| At4g12830 | alpha/beta-Hydrolases superfamily protein |
| At4g26950 | Protein of unknown function, DUF584 |
| At4g09350 | Chaperone DnaJ-domain superfamily protein |
| At5g52570 | B2, BCH2, BETA-OHASE 2, CHY2, beta-carotene hydroxylase 2 |
| At3g02730 | ATF1, TRXF1, thioredoxin F-type 1 |
| At2g23330 | transposable element gene |
| At2g37030 | SAUR-like auxin-responsive protein family |
| At5g24150 | SQE5, SQP1, FAD/NAD(P)-binding oxidoreductase family protein |
| At5g37790 | Protein kinase superfamily protein |
| At3g15770 | unknown protein; |
| At4g04830 | ATMSRB5, MSRB5, methionine sulfoxide reductase B5 |
| At5g04140 | FD-GOGAT, GLS1, GLU1, GLUS, glutamate synthase 1 |
| At1g23740 | Oxidoreductase, zinc-binding dehydrogenase family protein |
| At1g20880 | RNA-binding (RRM/RBD/RNP motifs) family protein |
| At4g10300 | RmlC-like cupins superfamily protein |
| At1g51805 | Leucine-rich repeat protein kinase family protein |
| At5g19220 | ADG2, APL1, ADP glucose pyrophosphorylase large subunit 1 |
| At5g25190 | Integrase-type DNA-binding superfamily protein |
| At1g11890 | ATSEC22, SEC22, Synaptobrevin family protein |
| At5g16800 | Acyl-CoA N-acyltransferases (NAT) superfamily protein |
| At1g28610 | GDSL-like Lipase/Acylhydrolase superfamily protein |
| At4g02420 | Concanavalin A-like lectin protein kinase family protein |
| At2g40860 | protein kinase family protein / protein phosphatase 2C ( PP2C) family protein |
| At4g31820 | ENP, MAB4, NPY1, Phototropic-responsive NPH3 family protein |
| At5g39610 | ANAC092, ATNAC2, ATNAC6, NAC2, NAC6, ORE1, NAC domain containing protein 6 |
| At1g44000 | unknown protein; |
| At1g66080 | unknown protein; |
| At4g39740 | Thioredoxin superfamily protein |
| At5g67160 | EPS1, HXXXD-type acyl-transferase family protein |
| At1g75280 | NmrA-like negative transcriptional regulator family protein |
| At3g26200 | CYP71B22, cytochrome P450, family 71, subfamily B, polypeptide 22 |
| At5g02020 | Encodes a protein involved in salt tolerance, names SIS (Salt Induced Serine rich). |
| At1g47270 | AtTLP6, TLP6, tubby like protein 6 |
| At3g13550 | CIN4, COP10, EMB144, FUS9, Ubiquitin-conjugating enzyme family protein |
| At5g56380 | F-box/RNI-like/FBD-like domains-containing protein |
| At5g39710 | EMB2745, Tetratricopeptide repeat (TPR)-like superfamily protein |
| At5g57910 | unknown protein; |
| At3g50750 | BEH1, BES1/BZR1 homolog 1 |
| At2g41980 | Protein with RING/U-box and TRAF-like domains |
| At1g75450 | ATCKX5, ATCKX6, CKX5, cytokinin oxidase 5 |
| At1g23410 | Ribosomal protein S27a / Ubiquitin family protein |
| At5g66250 | kinectin-related |
| At3g19850 | Phototropic-responsive NPH3 family protein |
| At3g05180 | GDSL-like Lipase/Acylhydrolase superfamily protein |
| At5g09660 | PMDH2, peroxisomal NAD-malate dehydrogenase 2 |
| At5g66640 | DAR3, DA1-related protein 3 |
| At1g69010 | BIM2, BES1-interacting Myc-like protein 2 |
| At1g80920 | J8, Chaperone DnaJ-domain superfamily protein |
| At2g19650 | Cysteine/Histidine-rich C1 domain family protein |
| At5g25180 | CYP71B14, cytochrome P450, family 71, subfamily B, polypeptide 14 |
| At3g50270 | HXXXD-type acyl-transferase family protein |
| At5g15230 | GASA4, GAST1 protein homolog 4 |
| At2g40610 | ATEXP8, ATEXPA8, ATHEXP ALPHA 1.11, EXP8, EXPA8, expansin A8 |
| At5g16990 | Zinc-binding dehydrogenase family protein |
| At5g18060 | SAUR-like auxin-responsive protein family |
| At2g37640 | ATEXP3, ATEXPA3, ATHEXP ALPHA 1.9, EXP3, Barwin-like endoglucanases superfamily protein |
| At2g25440 | AtRLP20, RLP20, receptor like protein 20 |
| At4g37560 | Acetamidase/Formamidase family protein |
| At5g05320 | FAD/NAD(P)-binding oxidoreductase family protein |
| At5g44400 | FAD-binding Berberine family protein |

Table S10. Significantly over-represented GO terms (FDR adjusted p-value <1E-6) in the 1084 functional targets of SDG8, determined using AgriGO TAIR10 version [5].

| GO_acc | Term | FDR adjusted pvalue |
| --- | --- | --- |
| GO:0050896 | response to stimulus | 2.20E-19 |
| GO:0006952 | defense response | 8.50E-19 |
| GO:0009987 | cellular process | 1.00E-18 |
| GO:0009814 | defense response, incompatible interaction | 2.90E-16 |
| GO:0006950 | response to stress | 2.90E-16 |
| GO:0008152 | metabolic process | 1.20E-15 |
| GO:0009627 | systemic acquired resistance | 1.20E-15 |
| GO:0045087 | innate immune response | 1.90E-15 |
| GO:0002376 | immune system process | 2.40E-15 |
| GO:0006955 | immune response | 2.40E-15 |
| GO:0044283 | small molecule biosynthetic process | 7.30E-15 |
| GO:0031347 | regulation of defense response | 7.30E-15 |
| GO:0051707 | response to other organism | 7.30E-15 |
| GO:0034641 | cellular nitrogen compound metabolic process | 7.30E-15 |
| GO:0046394 | carboxylic acid biosynthetic process | 7.30E-15 |
| GO:0016053 | organic acid biosynthetic process | 7.30E-15 |
| GO:0006796 | phosphate metabolic process | 8.60E-15 |
| GO:0006793 | phosphorus metabolic process | 8.60E-15 |
| GO:0080134 | regulation of response to stress | 1.80E-14 |
| GO:0048583 | regulation of response to stimulus | 2.50E-14 |
| GO:0044237 | cellular metabolic process | 3.00E-14 |
| GO:0006468 | protein amino acid phosphorylation | 3.70E-14 |
| GO:0043436 | oxoacid metabolic process | 7.80E-14 |
| GO:0019752 | carboxylic acid metabolic process | 7.80E-14 |
| GO:0006082 | organic acid metabolic process | 8.10E-14 |
| GO:0042180 | cellular ketone metabolic process | 1.70E-13 |
| GO:0006790 | sulfur metabolic process | 2.40E-13 |
| GO:0044281 | small molecule metabolic process | 4.00E-13 |
| GO:0009626 | plant-type hypersensitive response | 1.20E-12 |
| GO:0034050 | host programmed cell death induced by symbiont | 1.30E-12 |
| GO:0009607 | response to biotic stimulus | 2.00E-12 |
| GO:0045088 | regulation of innate immune response | 2.90E-12 |
| GO:0010363 | regulation of plant-type hypersensitive response | 2.90E-12 |
| GO:0007243 | protein kinase cascade | 3.20E-12 |
| GO:0050776 | regulation of immune response | 3.60E-12 |
| GO:0002682 | regulation of immune system process | 3.60E-12 |
| GO:0031348 | negative regulation of defense response | 3.80E-12 |
| GO:0048585 | negative regulation of response to stimulus | 3.80E-12 |
| GO:0016310 | phosphorylation | 4.30E-12 |
| GO:0012501 | programmed cell death | 4.80E-12 |
| GO:0080135 | regulation of cellular response to stress | 4.80E-12 |
| GO:0042221 | response to chemical stimulus | 4.80E-12 |
| GO:0043067 | regulation of programmed cell death | 6.80E-12 |
| GO:0000165 | MAPKKK cascade | 7.70E-12 |
| GO:0010941 | regulation of cell death | 1.00E-11 |
| GO:0009751 | response to salicylic acid stimulus | 2.00E-11 |
| GO:0009863 | salicylic acid mediated signaling pathway | 3.60E-11 |
| GO:0043687 | post-translational protein modification | 3.90E-11 |
| GO:0071446 | cellular response to salicylic acid stimulus | 3.90E-11 |
| GO:0051704 | multi-organism process | 4.30E-11 |
| GO:0009862 | systemic acquired resistance, salicylic acid mediated signaling pathway | 5.60E-11 |
| GO:0023052 | signaling | 6.80E-11 |
| GO:0033554 | cellular response to stress | 9.20E-11 |
| GO:0008219 | cell death | 1.30E-10 |
| GO:0006612 | protein targeting to membrane | 1.30E-10 |
| GO:0016265 | death | 1.30E-10 |
| GO:0006464 | protein modification process | 1.50E-10 |
| GO:0043412 | macromolecule modification | 1.60E-10 |
| GO:0044272 | sulfur compound biosynthetic process | 1.80E-10 |
| GO:0044271 | cellular nitrogen compound biosynthetic process | 1.90E-10 |
| GO:0019748 | secondary metabolic process | 2.30E-10 |
| GO:0010033 | response to organic substance | 2.70E-10 |
| GO:0006725 | cellular aromatic compound metabolic process | 2.70E-10 |
| GO:0007165 | signal transduction | 2.90E-10 |
| GO:0009697 | salicylic acid biosynthetic process | 3.60E-10 |
| GO:0009696 | salicylic acid metabolic process | 3.90E-10 |
| GO:0006519 | cellular amino acid and derivative metabolic process | 5.10E-10 |
| GO:0032787 | monocarboxylic acid metabolic process | 7.10E-10 |
| GO:0051716 | cellular response to stimulus | 1.20E-09 |
| GO:0009753 | response to jasmonic acid stimulus | 1.20E-09 |
| GO:0042440 | pigment metabolic process | 1.70E-09 |
| GO:0065007 | biological regulation | 1.90E-09 |
| GO:0006520 | cellular amino acid metabolic process | 1.90E-09 |
| GO:0044238 | primary metabolic process | 3.10E-09 |
| GO:0009617 | response to bacterium | 5.20E-09 |
| GO:0006629 | lipid metabolic process | 5.20E-09 |
| GO:0080010 | regulation of oxygen and reactive oxygen species metabolic process | 5.90E-09 |
| GO:0023046 | signaling process | 6.40E-09 |
| GO:0023060 | signal transmission | 6.40E-09 |
| GO:0042743 | hydrogen peroxide metabolic process | 1.30E-08 |
| GO:0009070 | serine family amino acid biosynthetic process | 1.50E-08 |
| GO:0019344 | cysteine biosynthetic process | 1.60E-08 |
| GO:0006534 | cysteine metabolic process | 1.70E-08 |
| GO:0010310 | regulation of hydrogen peroxide metabolic process | 1.80E-08 |
| GO:0051234 | establishment of localization | 1.80E-08 |
| GO:0019438 | aromatic compound biosynthetic process | 1.80E-08 |
| GO:0015994 | chlorophyll metabolic process | 2.00E-08 |
| GO:0044255 | cellular lipid metabolic process | 2.40E-08 |
| GO:0009867 | jasmonic acid mediated signaling pathway | 2.40E-08 |
| GO:0009308 | amine metabolic process | 2.40E-08 |
| GO:0071395 | cellular response to jasmonic acid stimulus | 2.40E-08 |
| GO:0044106 | cellular amine metabolic process | 2.80E-08 |
| GO:0009069 | serine family amino acid metabolic process | 3.00E-08 |
| GO:0006800 | oxygen and reactive oxygen species metabolic process | 3.00E-08 |
| GO:0000096 | sulfur amino acid metabolic process | 3.10E-08 |
| GO:0006811 | ion transport | 5.10E-08 |
| GO:0006810 | transport | 5.50E-08 |
| GO:0006778 | porphyrin metabolic process | 5.70E-08 |
| GO:0033013 | tetrapyrrole metabolic process | 6.20E-08 |
| GO:0009719 | response to endogenous stimulus | 1.10E-07 |
| GO:0051179 | localization | 1.30E-07 |
| GO:0031399 | regulation of protein modification process | 1.40E-07 |
| GO:0055082 | cellular chemical homeostasis | 1.40E-07 |
| GO:0035304 | regulation of protein amino acid dephosphorylation | 1.70E-07 |
| GO:0035303 | regulation of dephosphorylation | 2.10E-07 |
| GO:0051186 | cofactor metabolic process | 2.10E-07 |
| GO:0050801 | ion homeostasis | 2.10E-07 |
| GO:0019725 | cellular homeostasis | 2.70E-07 |
| GO:0006470 | protein amino acid dephosphorylation | 2.80E-07 |
| GO:0006873 | cellular ion homeostasis | 4.30E-07 |
| GO:0015979 | photosynthesis | 6.60E-07 |
| GO:0019252 | starch biosynthetic process | 9.00E-07 |
| GO:0000023 | maltose metabolic process | 9.50E-07 |

Table S11. Significantly over-represented KEGG pathways (FDR adjusted p-value <0.05) among the 1084 functional targets of SDG8 (while 158/1084 have KEGG annotation), determined using BioMaps function in VirtualPlant platform[6].

| Term | Observed Frequency | Expected Frequency | FDR adjusted  p-value |
| --- | --- | --- | --- |
| Energy Metabolism | 33 out of 158 genes, 20.9% | 313 out of 2905 genes, 10.8% | 0.00641 |
| Environmental Adaptation | 23 out of 158 genes, 14.6% | 172 out of 2905 genes, 5.9% | 0.00403 |
| Organismal Systems | 23 out of 158 genes, 14.6% | 172 out of 2905 genes, 5.9% | 0.00403 |
| Plant-pathogen interaction | 22 out of 158 genes, 13.9% | 143 out of 2905 genes, 4.9% | 0.00258 |
| Selenoamino acid metabolism | 9 out of 158 genes, 5.7% | 36 out of 2905 genes, 1.2% | 0.00655 |
| Sulfur metabolism | 8 out of 158 genes, 5.1% | 27 out of 2905 genes, 0.9% | 0.00641 |

Table S12. KEGG annotation [7] of the 33 “Energy Metabolism” pathway genes among the 1084 functional targets of SDG8.

| *Gene ID* | *KEGG annotation* | *KEGG pathway ID* |
| --- | --- | --- |
| AT4G01480 | Oxidative phosphorylation | ath00190 |
| AT4G23710 | Oxidative phosphorylation | ath00190 |
| AT1G53030 | Oxidative phosphorylation | ath00190 |
| AT4G05180 | photosynthesis | ath00195 |
| AT1G44575 | photosynthesis | ath00195 |
| AT4G05390 | photosynthesis | ath00195 |
| AT1G30380 | photosynthesis | ath00195 |
| AT2G27510 | photosynthesis | ath00195 |
| AT4G28660 | photosynthesis | ath00195 |
| AT5G64040 | photosynthesis | ath00195 |
| AT1G60950 | photosynthesis | ath00195 |
| AT3G08940 | Photosynthesis - antenna proteins | ath00196 |
| AT2G40100 | Photosynthesis - antenna proteins | ath00196 |
| AT1G45474 | Photosynthesis - antenna proteins | ath00196 |
| AT3G54050 | Carbon fixation in photosynthetic organisms | ath00710 |
| AT3G55800 | Carbon fixation in photosynthetic organisms | ath00710 |
| AT1G12900 | Carbon fixation in photosynthetic organisms | ath00710 |
| AT4G15530 | Carbon fixation in photosynthetic organisms | ath00710 |
| AT3G01850 | Carbon fixation in photosynthetic organisms | ath00710 |
| AT2G21330 | Carbon fixation in photosynthetic organisms | ath00710 |
| AT3G49160 | Carbon fixation in photosynthetic organisms | ath00710 |
| AT5G37600 | Nitrogen metabolism - Arabidopsis thaliana | ath00910 |
| AT1G70410 | Nitrogen metabolism - Arabidopsis thaliana | ath00910 |
| AT2G15620 | Nitrogen metabolism - Arabidopsis thaliana | ath00910 |
| AT5G35630 | Nitrogen metabolism - Arabidopsis thaliana | ath00910 |
| AT4G14680 | Sulfur metabolism | ath00920 |
| AT2G43750 | Sulfur metabolism | ath00920 |
| AT5G43780 | Sulfur metabolism | ath00920 |
| AT3G22890 | Sulfur metabolism | ath00920 |
| AT2G17640 | Sulfur metabolism | ath00920 |
| AT5G28020 | Sulfur metabolism | ath00920 |
| AT2G14750 | Sulfur metabolism | ath00920 |
| AT3G22460 | Sulfur metabolism | ath00920 |

Table S13. 54 genes whose H3K36me3 level increase in response to Carbon/light stimuli in WT but not in *sdg8-5*, and 9 genes whose H3K36me3 level increase in response to Carbon/light stimuli in the *sdg8-5* mutant.

| **A) Genes whose H3K36me3 level increase in response to C/L in WT** | |
| --- | --- |
| **Gene ID** | **Tair10 Annotation** |
| At4g36120 | Plant protein of unknown function (DUF869) |
| At1g69610 | Protein of unknown function (DUF1666) |
| At5g64510 | unknown protein |
| At1g33110 | MATE efflux family protein |
| At5g53420 | CCT motif family protein |
| At5g38420 | Ribulose bisphosphate carboxylase (small chain) family protein |
| At4g37930 | SHM1, SHMT1, STM, serine transhydroxymethyltransferase 1 |
| At4g12830 | alpha/beta-Hydrolases superfamily protein |
| At1g34060 | Pyridoxal phosphate (PLP)-dependent transferases superfamily protein |
| At2g04039 | unknown protein; |
| At2g25510 | unknown protein |
| At3g28070 | nodulin MtN21 /EamA-like transporter family protein |
| At4g13810 | AtRLP47, RLP47, receptor like protein 47 |
| At4g04020 | FIB, fibrillin |
| At1g80130 | Tetratricopeptide repeat (TPR)-like superfamily protein |
| At2g29120 | ATGLR2.7, GLR2.7, GLR2.7, glutamate receptor 2.7 |
| At5g43630 | TZP, zinc knuckle (CCHC-type) family protein |
| At5g24120 | ATSIG5, SIG5, SIGE, sigma factor E |
| At3g21760 | HYR1, UDP-Glycosyltransferase superfamily protein |
| At4g36640 | Sec14p-like phosphatidylinositol transfer family protein |
| At3g44450 | unknown protein; |
| At1g13470 | Protein of unknown function (DUF1262) |
| At4g12290 | Copper amine oxidase family protein |
| At4g10770 | ATOPT7, OPT7, oligopeptide transporter 7 |
| At5g43150 | unknown protein; |
| At2g23840 | HNH endonuclease |
| At1g44575 | NPQ4, PSBS, Chlorophyll A-B binding family protein |
| At1g64970 | G-TMT, TMT1, VTE4, gamma-tocopherol methyltransferase |
| At5g54610 | ANK, ankyrin |
| At1g22160 | Protein of unknown function (DUF581) |
| At5g59050 | unknown protein |
| At4g33040 | Thioredoxin superfamily protein |
| At5g06510 | NF-YA10, nuclear factor Y, subunit A10 |
| At3g11080 | AtRLP35, RLP35, receptor like protein 35 |
| At1g10690 | unknown protein; |
| At5g02180 | Transmembrane amino acid transporter family protein |
| At3g56290 | unknown protein; |
| At2g20560 | DNAJ heat shock family protein |
| At3g22370 | AOX1A, ATAOX1A, alternative oxidase 1A |
| At4g21990 | APR3, ATAPR3, PRH-26, PRH26, APS reductase 3 |
| At3g55630 | ATDFD, DFD, DHFS-FPGS homolog D |
| At2g31380 | STH, salt tolerance homologue |
| At5g25120 | CYP71B11, cytochrome p450, family 71, subfamily B, polypeptide 11 |
| At4g23320 | CRK24, cysteine-rich RLK (RECEPTOR-like protein kinase) 24 |
| At5g52640 | ATHS83, AtHsp90-1, ATHSP90.1, HSP81-1, HSP81.1, HSP83, HSP90.1, heat shock protein 90.1 |
| At1g13080 | CYP71B2, cytochrome P450, family 71, subfamily B, polypeptide 2 |
| At4g17670 | Protein of unknown function (DUF581) |
| At1g61300 | LRR and NB-ARC domains-containing disease resistance protein |
| At2g26150 | ATHSFA2, HSFA2, heat shock transcription factor A2 |
| At5g58770 | Undecaprenyl pyrophosphate synthetase family protein |
| At5g61270 | PIF7, phytochrome-interacting factor7 |
| At5g38430 | Ribulose bisphosphate carboxylase (small chain) family protein |
| At3g12580 | ATHSP70, HSP70, heat shock protein 70 |
| At5g62680 | Major facilitator superfamily protein |
| **B) Genes whose H3K36me3 level increase in response to C/L in sdg8-5** | |
| At4g39970 | Haloacid dehalogenase-like hydrolase (HAD) superfamily protein |
| At3g23810 | ATSAHH2, SAHH2, S-adenosyl-l-homocysteine (SAH) hydrolase 2 |
| At1g26230 | TCP-1/cpn60 chaperonin family protein |
| At3g19480 | D-3-phosphoglycerate dehydrogenase |
| At5g49450 | AtbZIP1, bZIP1, basic leucine-zipper 1 |
| At5g49448 | CPuORF4, conserved peptide upstream open reading frame 4 |
| At3g02830 | ZFN1, zinc finger protein 1 |
| At3g63140 | CSP41A, chloroplast stem-loop binding protein of 41 kDa |
| At5g20070 | ATNUDT19, ATNUDX19, NUDX19, nudix hydrolase homolog 19 |

Table S14. Significantly over-represented biological processes GO terms (FDR adjusted p-value <0.01) among the 54 genes whose H3K36me3 level increase in response to Carbon/light stimuli in WT but not in *sdg8-5* mutant, determined using BioMaps function in VirtualPlant platform[6].

| **GO terms** | **GO annotation** | **FDR adjusted p-value** |
| --- | --- | --- |
| GO:0009628 | response to abiotic stimulus | 9.70E-06 |
| GO:0009314 | response to radiation | 5.32E-05 |
| **GO:0009416** | **response to light stimulus** | **5.32E-05** |
| GO:0050896 | response to stimulus | 0.000696 |
| GO:0009266 | response to temperature stimulus | 0.000895 |
| GO:0009573 | chloroplast ribulose bisphosphate carboxylase complex | 0.00294 |
| GO:0048492 | ribulose bisphosphate carboxylase complex | 0.00294 |
| GO:0009644 | response to high light intensity | 0.00411 |
| GO:0010218 | response to far red light | 0.00411 |
| **GO:0015977** | **carbon fixation** | **0.00411** |
| GO:0016984 | ribulose-bisphosphate carboxylase activity | 0.00411 |
| GO:0071704 | organic substance metabolic process | 0.00411 |
| GO:0009637 | response to blue light | 0.00502 |
| GO:0009408 | response to heat | 0.00513 |
| GO:0010114 | response to red light | 0.00513 |

Table S15. Gene IDs and primer sequences for ChIP-PCR validation.

| gene name | At Locus ID | ChIP-PCR primer sequence |
| --- | --- | --- |
| AT1G56220 | AT1G56220 | Forward TCAATCGGAAGCTGGAAGTG |
|  |  | Reverse TTGGTAACCCGGTGGTTTAAT |
| COL4 | AT5G24930 | Forward TGGCTGTTACTTCCCAATCC |
|  |  | Reverse CAGTGTTCATCTCCGGACTATC |
| LAZ5 | AT5G44870 | Forward GACCTCAGCTGGAATCACATAC |
|  |  | Reverse GCGATTTCCGATTCCTCTATCC |
| MAF1 | AT1G77080 | Forward CTCATCGACAAAGCTCGACAA |
|  |  | Reverse TCACCGGAGGAAGAGTCATAG |
| PIL5 | AT2G20180 | Forward ACCGAAGCTTCTTCCTTCTATG |
|  |  | Reverse CATCGTCACGGAGAGGATAATG |
| PGRL1B | AT4G11960 | Forward GACTTGTGGACCTCCTTGATTAT |
|  |  | Reverse CACAGTCCGAGAGCAACAA |
| FLC | AT5G10140 | 1) Forward GAACCCAAACCTGAGGATCAA |
|  |  | 1) Reverse GGTGACTTGTCGGCTACTTT |
|  |  | 2) Forward TAGGTCCAGCCTTGGAATTG |
|  |  | 2) Reverse GTAACTAAGGGTTCCACGTTCT |
|  |  | 3) Forward GTGACTAGAGCCAAGAAGGTAAG |
|  |  | 3) Reverse TGAGTTCGGTCTGCAACAA |
|  |  | 4) Forward CCTTAAATCGGCGGTTGAAATC |
|  |  | 4) Reverse TACAAACGCTCGCCCTTATC |
| actin1 | AT2G37620 | Forward CAACAATTGGGATGACATGGAG |
|  |  | Reverse GGTGCCTCGGTAAGTAGAATAG |
| RNA helicase | AT1G58050 | Forward CCAAGGCAAGTGACAACAAC |
|  |  | Reverse CGGAGGTTCCAAAGCCTAAA |

Table S16. Significantly over-represented KEGG pathways (FDR adjusted p-value <0.05) among the 728 SDG8 direct targets (while 172/728 have KEGG annotation), determined using BioMaps function in VirtualPlant platform[6].

| **Term** | **Observed Frequency** | **Expected Frequency** | **FDR adjusted p-value** |
| --- | --- | --- | --- |
| Ribosome | 42 out of 172 genes, 24.4% | 7.2% | 1.32E-09 |
| Translation | 49 out of 172 genes, 28.5% | 14.8% | 0.000294 |
| Energy Metabolism | 38 out of 172 genes, 22.1% | 10.7% | 0.000646 |
| Photosynthesis | 12 out of 172 genes, 7% | 1.5% | 0.000761 |
| Photosynthesis - antenna proteins | 8 out of 172 genes, 4.7% | 0.7% | 0.00223 |

Table S17. KEGG annotation [7] of the 38 “Energy Metabolism” pathway genes among the 728 direct targets of SDG8.

| **Gene ID** | **KEGG annotation** | **KEGG pathway** |
| --- | --- | --- |
| AT1G23310 | Carbon fixation in photosynthetic organisms | ath00710 |
| AT1G32060 | Carbon fixation in photosynthetic organisms | ath00710 |
| AT1G42970 | Carbon fixation in photosynthetic organisms | ath00710 |
| AT1G67090 | Carbon fixation in photosynthetic organisms | ath00710 |
| AT2G36460 | Carbon fixation in photosynthetic organisms | ath00710 |
| AT3G52930 | Carbon fixation in photosynthetic organisms | ath00710 |
| AT3G55800 | Carbon fixation in photosynthetic organisms | ath00710 |
| AT3G60750 | Carbon fixation in photosynthetic organisms | ath00710 |
| AT4G38970 | Carbon fixation in photosynthetic organisms | ath00710 |
| AT1G58180 | nitrogen metabolism | ath00910 |
| AT5G37600 | nitrogen metabolism | ath00910 |
| AT1G15690 | Oxidative phosphorylation | ath00190 |
| AT2G18960 | Oxidative phosphorylation | ath00190 |
| AT3G15352 | Oxidative phosphorylation | ath00190 |
| AT3G52730 | Oxidative phosphorylation | ath00190 |
| AT1G06680 | Photosynthesis | ath00195 |
| AT1G08380 | Photosynthesis | ath00195 |
| AT1G20340 | Photosynthesis | ath00195 |
| AT1G31330 | Photosynthesis | ath00195 |
| AT1G67740 | Photosynthesis | ath00195 |
| AT1G79040 | Photosynthesis | ath00195 |
| AT2G30570 | Photosynthesis | ath00195 |
| AT4G03280 | Photosynthesis | ath00195 |
| AT4G05180 | Photosynthesis | ath00195 |
| AT4G28750 | Photosynthesis | ath00195 |
| AT5G64040 | Photosynthesis | ath00195 |
| AT5G66570 | Photosynthesis | ath00195 |
| AT1G61520 | Photosynthesis - antenna proteins | ath00196 |
| AT2G05070 | Photosynthesis - antenna proteins | ath00196 |
| AT2G05100 | Photosynthesis - antenna proteins | ath00196 |
| AT3G47470 | Photosynthesis - antenna proteins | ath00196 |
| AT3G54890 | Photosynthesis - antenna proteins | ath00196 |
| AT3G61470 | Photosynthesis - antenna proteins | ath00196 |
| AT4G10340 | Photosynthesis - antenna proteins | ath00196 |
| AT5G54270 | Photosynthesis - antenna proteins | ath00196 |
| AT3G22890 | sulfur metabolism | ath00920 |
| AT3G61440 | sulfur metabolism | ath00920 |
| AT3G53260 | NA | NA |
